# Supplementary material for: ER stress-mediated impairment of hepatic lipid export is associated with steatosis in AKI-induced remote liver injury
Source: Sci Rep. 2026 Jun 29;16:29943. doi: 10.1038/s41598-026-59445-3 (PMC13616736; doi:10.1038/s41598-026-59445-3)
Supplement: Supplementary file 1 [file 41598_2026_59445_MOESM1_ESM.pdf]

Supplemental Materials

Experimental method:

Serum Biochemistry

Blood was collected via cardiac puncture under deep anesthesia. Serum was separated by centrifugation at 3,000 × g for 10 minutes at 4°C. Serum creatinine and blood urea nitrogen (BUN) were measured to assess renal function. Hepatic injury was evaluated by serum alanine aminotransferase (ALT) and aspartate aminotransferase (AST) activity. All biochemical parameters were analyzed using commercial colorimetric assay kits according to the manufacturer's protocols.

Histopathological Analysis

Kidney and liver tissues were fixed in 10% neutral buffered formalin for 24 hours, processed, and embedded in paraffin. Sections (4 µm) were stained with hematoxylin and eosin (H&E) for morphological evaluation. Renal tubular injury was assessed based on tubular necrosis, cast formation, and brush border loss. Hepatic injury was evaluated by examining hepatocyte architecture, sinusoidal congestion, and inflammatory infiltration.

For lipid accumulation analysis, frozen liver sections (8 µm) were prepared using optimal cutting temperature (OCT) compound. Sections were stained with commercially available Oil Red O staining kit (Abcam). Oil Red O–positive area was evaluated with a fixed intensity threshold, averaged across 5 sections per animal (5 randomly selected fields per section); the animal was treated as the experimental unit (n = 5 per group).

Quantitative Proteomics

Peptides were analyzed using a Q Exactive HF-X hybrid quadrupole-Orbitrap mass spectrometer (Thermo Fisher Scientific) coupled to an Easy-nLC 1200 system (Thermo Fisher Scientific). Samples were loaded onto an Acclaim PepMap 100 trap column (75 µm × 2 cm, C18, 3 µm, 100 Å) and separated on an Easy-Spray analytical column (75 µm × 25 cm, C18, 100 Å) at a flow rate of 300 nL/min. A 120-minute gradient was employed: mobile phase B (0.1% formic acid in 90% acetonitrile) was increased from 2% to 28% over 100 min, then to 40% over 10 min, followed by a wash at 90% B for 15 min. Mobile phase A consisted of 0.1% formic acid in water.

The mass spectrometer was operated with a spray voltage of 2 kV and capillary temperature of 300°C. Full MS scans were acquired at a resolution of 60,000 (AGC target 3×10^6, maximum injection time 50 ms) over a mass range of 400–1,600 m/z. Data-dependent MS/MS acquisition selected the top 30 precursor ions with an isolation window of 1.6 m/z, resolution of 15,000 (AGC target 1×10^4, maximum injection time 50 ms), and dynamic exclusion of 25 s.

Raw data were processed using MaxQuant (v2.0) with the Andromeda search engine against the UniProt mouse proteome database. Search parameters included trypsin digestion with up to two missed cleavages, precursor mass tolerance of 10 ppm, and fragment mass tolerance of 20 ppm. Variable modifications included methionine oxidation and N-terminal acetylation; carbamidomethylation of cysteine was set as a fixed modification. Label-free quantification (LFQ) was enabled with match-between-runs. A false discovery rate (FDR) of 1% was applied at both peptide and protein levels.

Data Analysis

LFQ proteomics data were processed using a custom Snakemake pipeline (v4.5.2).

Contaminants, reverse sequences, and proteins identified only by site were excluded. Proteins

with fewer than two unique peptides were removed. To ensure reliable quantification, proteins were required to have valid intensity values in at least 70% of samples within each experimental group. Missing values were imputed using the QRILC method with a quantile threshold of 0.01, appropriate for left-censored proteomics data. LFQ intensities were normalized using the MaxLFQ algorithm. All samples were processed in a single batch, and no batch-effect correction was applied.

Differential protein abundance was assessed using DEqMS (v1.28.0), which extends the limma framework by incorporating peptide count information to more accurately estimate protein variance. Statistical significance was determined using an adjusted P-value threshold of 0.05 (Benjamini-Hochberg correction) and an absolute $\log_2$ fold-change threshold of 1.

Pathway enrichment analysis employed a hybrid approach combining over-representation analysis (ORA) and gene set enrichment analysis (GSEA). ORA was applied to significantly differentially abundant proteins, while GSEA was performed on all detected proteins ranked by signed significance score ($-\log_{10}(\text{P-value}) \times \text{sign}(\log_2 FC)$). Enrichment was tested against multiple databases: Gene Ontology (Biological Process, Molecular Function, Cellular Component), KEGG (Kanehisa et al., 2025; Kanehisa and Goto, 2000), Reactome, MSigDB Hallmark, and WikiPathways. Semantically similar GO terms were collapsed using the simplify function (similarity cutoff = 0.7, Wang method) from clusterProfiler (v4.18.4). Pathway activity was inferred using PROGENy, a footprint-based method that estimates the activity of 14 signaling pathways from protein expression changes using a curated set of downstream response genes. Pathways with a false discovery rate (FDR) below 0.25 were considered statistically significant, consistent with standard GSEA practice; this threshold is distinct from the adjusted $P < 0.05$ applied to individual differentially abundant proteins.

Western Blot Analysis

Liver tissues were flash-frozen in liquid nitrogen and stored at −80 °C until protein extraction. Homogenization was carried out using either T-PER™ Tissue Protein Extraction Reagent (Thermo Fisher Scientific) in combination with the FastPrep-24™ 5G bead-beating system (MP Biomedicals), following the manufacturer's protocol, or a sucrose-based buffer (300 mM sucrose, 50 mM Tris·HCl pH 7.4, 1 mM EDTA, 1 mM EGTA, 1 mM Na_3VO_4, 50 mM NaF, 1 mM DTT, 1 mM PMSF, 1 µg/mL aprotinin, 4 µg/mL leupeptin, and PhosStop; Roche). Proteins were separated by SDS-PAGE (standard or 4–15% Criterion TGX Stain-Free gels; Bio-Rad) and transferred to PVDF membranes. Membranes were blocked with 5% nonfat dry milk in TBS-T, incubated overnight at 4 °C with primary antibodies, and subsequently with secondary antibodies for 1 h at room temperature. Detection was performed using Western Lightning Plus-ECL (PerkinElmer) and imaged with the ChemiDoc Imaging System (Bio-Rad). See Supplementary Table S4 for complete antibody information. And see Supplementary Figure S5 for the original, unprocessed, full-length membranes for every panel.

Immunohistochemistry

Paraffin-embedded liver sections (4 µm) were deparaffinized and rehydrated through graded alcohols. Antigen retrieval was performed using citrate buffer (pH 6.0) in a pressure cooker for 15 minutes. Endogenous peroxidase activity was quenched with 3% H2O2 for 10 minutes. Sections were blocked with 5% normal serum for 1 hour, then incubated overnight at 4°C with primary antibodies against CD68 (1:200) or F4/80 (1:200). Detection was performed using a polymer-based HRP detection system with DAB chromogen. Sections were counterstained with hematoxylin and mounted. Positive cells were evaluated from 5 random high-power fields per section.

Quantitative Reverse-Transcription PCR

A small piece of freshly harvested liver tissue was immediately kept in RNAlater solution (Invitrogen, USA) according to the manufacture's protocol. Total RNA was isolated using TRIzol™ Reagent (Invitrogen, USA) according to the manufacturer's instructions and quantified by NanoDrop One system (Thermo Fisher Scientific, USA). Then, the RNA was reverse transcribed with iScript Reverse Transcription Supermix (Bio-Rad). qPCR was performed on a CFX96 real-time system (Bio-Rad). The primers used in this study were listed in Supplementary Table S5.

XBP1 Splicing Assay

Activation of the IRE1α branch of the unfolded protein response was assessed by quantifying spliced Xbp1 (Xbp1s) mRNA levels. Under ER stress, activated IRE1α excises a 26-nucleotide intron from Xbp1 mRNA, generating the transcriptionally active Xbp1s isoform. To specifically detect this spliced variant, qPCR was performed using primers designed to span the splice junction (Forward: 5′-CTGAGTCCGAATCAGGTGCAG-3′; Reverse: 5′-GGTCCATGGGAAGATGTTCTGG-3′), which selectively amplify Xbp1s but not unspliced Xbp1u.

Supplementary Methods: Clinical Cohort Analyses

Cohort Derivation: MIMIC-IV

The MIMIC-IV (version 3.1) database contained 94,458 ICU admissions. Patients were excluded sequentially as follows: ICU length of stay shorter than 24 hours (n = 18,786); pre-existing liver disease identified by ICD-9 code 571.x or ICD-10 codes K70 through K77 (n = 11,584); prior

liver transplantation (n = 202); chronic kidney disease stage 3 or higher, defined by diagnosis codes or estimated glomerular filtration rate below 60 mL/min/1.73 m2 calculated using the CKD-EPI 2021 equation (n = 13,577 by diagnosis codes; n = 4,872 by eGFR); missing baseline serum creatinine within seven days of ICU admission (n = 830); missing baseline alanine aminotransferase or aspartate aminotransferase (n = 28,346); and baseline ALT or AST exceeding twice the upper limit of normal (>80 U/L; n = 3,557). After all exclusions, 12,704 patients remained (3,803 with AKI and 8,901 without AKI). A CONSORT-style flow diagram is presented in Figure 7.

AKI was classified using the kidney disease: Improving Global Outcomes (KDIGO) serum creatinine criteria. Baseline creatinine was defined as the lowest value recorded within the first seven days of ICU admission. Stage 1 required a rise of 0.3 mg/dL or more within 48 hours, or a 1.5-fold increase above baseline within seven days. Thresholds for Stages 2 and 3 were 2.0-fold and 3.0-fold increases above baseline, respectively; Stage 3 was also assigned when creatinine exceeded 4.0 mg/dL or renal replacement therapy was initiated. Urine output criteria were not used for primary staging.

Propensity Score Matching and Covariate Balance: MIMIC-IV

Propensity scores were estimated using logistic regression with the following covariates: age, sex, non-renal Sequential Organ Failure Assessment (SOFA) score, Sepsis-3 status, diabetes, vasopressor use, and admission category (emergency, surgical, elective, or other). Matching was performed at a 1:1 ratio using nearest-neighbor matching without replacement, with a caliper width of 0.2 standard deviations of the logit-transformed propensity score (caliper = 0.1399). Of 3,803 eligible AKI patients, 3,498 (92.0%) were successfully matched to 3,498 controls, yielding

a final analytic cohort of 6,996 patients. The remaining 305 AKI patients fell outside the caliper and were excluded.

Covariate balance was assessed using standardized mean differences (SMD), with a threshold of less than 0.1 indicating adequate balance. All nine matching covariates achieved excellent balance after matching (Table S6). The maximum SMD decreased from 0.593 (non-renal SOFA score) before matching to 0.037 (age) after matching. Individual covariate SMDs after matching were as follows: age, 0.037; sex, 0.009; non-renal SOFA score, 0.010; sepsis, 0.016; diabetes, 0.024; vasopressor use, 0.013; emergency admission, 0.034; other admission category, 0.018; and surgical admission, 0.016. ICU length of stay, hospital mortality, statin use, acetaminophen exposure, and mechanical ventilation were not included in the propensity model because they represent post-admission exposures or outcomes that may lie on the causal pathway between AKI and hepatic dysfunction. Baseline demographics of the matched cohort are reported in Table 3 of the main manuscript.

The matched AKI cohort comprised 2,196 patients with KDIGO Stage 1 (62.8%), 866 with Stage 2 (24.8%), and 436 with Stage 3 (12.5%). Both groups were comparable in age (63.1 versus 63.7 years; SMD = 0.037), sex distribution (55.3% versus 54.8% male; SMD = 0.009), non-renal SOFA score (median 4 in both groups; SMD = 0.010), sepsis prevalence (47.4% versus 48.2%; SMD = 0.016), diabetes (28.6% versus 27.6%; SMD = 0.024), and vasopressor use (42.7% versus 42.1%; SMD = 0.013).

Outcome Ascertainment: MIMIC-IV

Peak values for ALT, AST, total bilirubin, and alkaline phosphatase were extracted within 72 hours of AKI onset for AKI patients (or the matched reference time for controls). Albumin nadir

was determined over the same window. Clinically significant liver injury was defined as peak ALT exceeding twice the upper limit of normal (>80 U/L).

Between-group comparisons used the Mann-Whitney U test. Severity-dependent relationships across KDIGO stages were evaluated using Kruskal-Wallis and Jonckheere-Terpstra trend tests. Temporal trajectories of liver markers were modeled with linear mixed-effects models incorporating log-transformed values, a time-by-group interaction term, and a random intercept per patient. Multivariable logistic regression estimated the adjusted odds of liver injury, controlling for age, sex, non-renal SOFA score, sepsis status, vasopressor use, diabetes, statin use, acetaminophen exposure, and mechanical ventilation.

Sensitivity Analyses: MIMIC-IV

Four pre-specified sensitivity analyses were conducted: (1) stricter baseline exclusion requiring ALT and AST both below the upper limit of normal (40 U/L); (2) analysis of the full unmatched cohort (n = 12,704) with multivariable adjustment; (3) liver injury defined by AST rather than ALT (AST >80 U/L); and (4) exclusion of patients who received potentially hepatotoxic antibiotics during the ICU stay.

Cohort Derivation: eICU-CRD

The eICU Collaborative Research Database (eICU-CRD, version 2.0) contained 200,859 ICU admissions from 208 hospitals across the United States during 2014 and 2015. Patients were excluded sequentially: age younger than 18 years (n = 530); pre-existing end-stage renal disease or chronic dialysis, identified from diagnosis codes and past medical history fields (n = 8,216); ICU length of stay shorter than 24 hours (n = 65,539); pre-existing liver cirrhosis (n = 4,101); and absence of serum creatinine data (n = 5,347). The final analytic cohort comprised 117,126

ICU stays, of which 24,905 (21.3%) met KDIGO creatinine criteria for AKI: 14,920 Stage 1 (12.7%), 1,961 Stage 2 (1.7%), and 8,024 Stage 3 (6.9%).

Baseline creatinine in the eICU-CRD analysis was defined as the first serum creatinine measured within 24 hours of ICU admission, with fallback to the lowest value within 48 hours if no measurement was available in the first 24 hours. AKI staging followed the same KDIGO creatinine thresholds described for MIMIC-IV. Renal replacement therapy initiation was identified from the treatment table and APACHE assessment variables to capture additional Stage 3 patients.

Lipid Measurements: eICU-CRD

Serum lipid measurements (triglycerides, total cholesterol, HDL cholesterol, and LDL cholesterol) were extracted from the laboratory table using verified labName strings. Measurement timestamps were derived from the labResultOffset variable, expressed as minutes from ICU admission. Pre-admission measurements (negative offset values) were excluded from the primary analysis, yielding 80,690 post-admission lipid measurements. After outlier removal (triglycerides below 10 mg/dL or above 10,000 mg/dL; total cholesterol below 20 mg/dL or above 1,000 mg/dL; LDL below 1 mg/dL or above 500 mg/dL; 17 values excluded total), 102,725 clean lipid measurements from 28,519 patients were available for analysis. Among these, 22,727 patients had at least one triglyceride measurement (5,956 AKI; 16,771 non-AKI), 16,205 had total cholesterol, 16,226 had HDL, and 12,017 had LDL data.

Statistical Analysis: eICU-CRD

Primary comparisons of lipid levels between AKI and non-AKI groups used Mann-Whitney U tests, given non-normal distributions. Effect sizes were reported as rank-biserial correlation

coefficients and Cohen's d. Stratified analyses by KDIGO stage used Kruskal-Wallis tests with post-hoc Bonferroni-corrected pairwise comparisons. Multivariable linear regression of log-transformed lipid values was performed with adjustment for age, sex, APACHE IV score, body mass index (BMI), and diabetes status. Subgroup analyses examined the consistency of lipid associations across sepsis status, surgical versus medical admission, sex, and diabetes.

Temporal analysis used measurements binned into six time windows (0-24h, 24-48h, 48-72h, 72-96h, 4-7 days, and >7 days). For patients with two or more triglyceride measurements (n = 4,654), a linear mixed-effects model was fitted using restricted maximum likelihood (REML) estimation. Log-transformed triglycerides served as the outcome. Fixed effects included time (days), AKI status, and their interaction, with adjustment for age, sex, APACHE IV score, BMI, and diabetes; a random intercept was specified for each patient. A secondary AKI definition based on diagnosis codes was used for sensitivity analysis, confirming concordant results (median TG 132 versus 111 mg/dL; p = 8.3 x 10-48).

Cross-Database Comparison

Triglyceride levels were compared between the eICU-CRD primary analysis (n = 22,727) and an exploratory subset from MIMIC-IV with available triglyceride data (AKI n = 175; control n = 55). Both databases showed significantly elevated triglycerides in the AKI group (eICU-CRD: 124 versus 111 mg/dL, p = 4.9 x 10-40; MIMIC-IV: 269 versus 188 mg/dL, p < 0.001). Absolute triglyceride values differed between databases, likely reflecting differences in measurement timing (median first triglyceride measurement at 16 to 36 hours post-admission in eICU-CRD versus peri-AKI onset sampling in MIMIC-IV), population characteristics (multi-center versus single-center), and the smaller MIMIC-IV lipid subset. The direction and statistical significance of the AKI-associated triglyceride elevation were concordant across both databases.

Software

All clinical analyses were performed in Python 3.9.6 using the following packages: pandas 2.3.3 for data manipulation, scipy 1.13.1 for non-parametric statistical tests, statsmodels 0.14.6 for regression modeling and mixed-effects models, scikit-learn 1.6.1 for propensity-score estimation, tableone 0.9.6 for demographic table generation, matplotlib 3.9.4 and seaborn 0.13.2 for visualization, and google-cloud-bigquery 3.40.1 for database access. Propensity score estimation and matching for the MIMIC-IV analysis used scikit-learn logistic regression with greedy nearest-neighbor matching implemented via custom scripts. A two-sided p-value below 0.05 was considered statistically significant throughout.

Supplementary Figure Legends

Supplementary Figure 1. AKI Validation and Inflammatory Profiling

(A) Plasma creatinine levels in Sham and AKI mice at 24 hours post-surgery, confirming successful induction of kidney injury. Data are presented as mean ± SD. *$p < 0.05$ by unpaired Student's t-test. (B) Cytokine array analysis of liver tissue samples from Sham and AKI mice. Representative array membranes showing relative abundance of inflammatory cytokines and chemokines. Overall cytokine levels are reduced in AKI compared to Sham, consistent with the paradoxical suppression of inflammatory output described in the main text. (C) Densitometric quantification of selected pro-inflammatory cytokines (TNF-α, IL-1β, IL-6) from the cytokine array membranes shown in (B). Values are normalized to Sham controls and expressed as relative blot density. Despite TLR4/MyD88 activation in AKI livers, circulating levels of these canonical

inflammatory mediators trended lower in AKI animals, though differences did not reach statistical significance (ns, not significant). Data are presented as mean ± SD; unpaired Student's t-test. Abbreviations: AKI, acute kidney injury; IL-1β, interleukin-1 beta; IL-6, interleukin-6; ns, not significant; SD, standard deviation; TLR4, Toll-like receptor 4; MyD88, myeloid differentiation primary response 88; TNF-α, tumor necrosis factor alpha.

Supplementary Figure 2. Transcript-Level Validation of Key Signaling and Metabolic Genes

(A) Quantitative RT-PCR analysis of TLR4 co-receptors. CD14 transcript levels are significantly upregulated in AKI livers, while MD-2 shows no significant change. (B) Quantitative RT-PCR analysis of macrophage markers. F4/80 expression is significantly reduced, CD68 is significantly increased, and CD163 is significantly upregulated in AKI livers. (C) Quantitative RT-PCR analysis of fatty acid handling genes. Fabp1 expression is significantly reduced, while Acox1 shows a compensatory increase in AKI livers. Expression normalized to GAPDH and presented relative to Sham controls. Data are mean ± SEM; n=5 per group. *$p < 0.05$ by unpaired two-tailed Student’s t-test. Abbreviations: AKI, acute kidney injury; CD14/CD68/CD163, cluster of differentiation 14/68/163; F4/80, EGF-like module-containing mucin-like hormone receptor-like 1; GAPDH, glyceraldehyde-3-phosphate dehydrogenase; MD-2, myeloid differentiation factor 2; RT-PCR, reverse-transcription polymerase chain reaction; SEM, standard error of the mean; TLR4, Toll-like receptor 4.

Supplementary Figure 3. Comprehensive Pathway Enrichment Analysis

(A) MSigDB Hallmark gene set enrichment analysis (GSEA) showing enriched pathways in AKI livers. Dot size represents gene count; color indicates adjusted p-value. (B) KEGG pathway

enrichment analysis showing additional dysregulated pathways in AKI livers, including xenobiotic metabolism and steroid hormone biosynthesis. Dot size represents gene count; color indicates adjusted p-value. Pathway annotations in panel B were obtained from the KEGG database (Kanehisa et al., 2025; Kanehisa and Goto, 2000). Abbreviations: AKI, acute kidney injury; GSEA, gene set enrichment analysis; KEGG, Kyoto Encyclopedia of Genes and Genomes; MSigDB, Molecular Signatures Database.

Supplementary Figure 4. Cross-Database Comparison of Serum Triglyceride Levels in Acute Kidney Injury

(A) Forest plot showing percent change in serum triglycerides associated with AKI across two independent ICU databases (eICU-CRD, multi-center, 208 hospitals; MIMIC-IV, single-center). eICU-CRD analyses include unadjusted and covariate-adjusted estimates (adjusted for age, sex, APACHE IV score, BMI, and diabetes) along with KDIGO stage-specific effects. Error bars represent 95% confidence intervals. (B) Median triglyceride levels with interquartile range in AKI versus non-AKI groups in each database. Solid bars indicate eICU-CRD; hatched bars indicate MIMIC-IV. ***$p < 0.001$. (C) Lipid dissociation pattern in eICU-CRD: percent change in four serum lipid markers (triglycerides, total cholesterol, HDL, LDL) in AKI compared with non-AKI patients. Triglycerides were elevated (+11.7%) while total cholesterol, HDL, and LDL were all reduced (range: -10.3% to -15.4%). Abbreviations: AKI, acute kidney injury; APACHE IV, Acute Physiology and Chronic Health Evaluation IV; BMI, body mass index; eICU-CRD, eICU Collaborative Research Database; HDL, high-density lipoprotein; KDIGO, Kidney Disease: Improving Global Outcomes; LDL, low-density lipoprotein; MIMIC-IV, Medical Information Mart for Intensive Care IV.

Supplementary Figure 5. Uncropped Western blot membranes corresponding to cropped panels in main figures. Western blot membranes are shown for all proteins displayed in the main figures. Panels show: (A) GRP78, CHOP and GAPDH loading control corresponding to Fig. 4B; (B) TLR4, MyD88, and GAPDH loading control corresponding to Fig. 5A. Red dashed boxes outline the regions shown in the respective main figure panels. Molecular weight markers (kDa) are indicated on the left side of each membrane. Lanes correspond to Sham (n = 5) and AKI (n = 5) samples. Images were exported directly from Bio-Rad Image Lab software; brightness and contrast adjustments were applied uniformly across each entire image without region-specific or non-linear modification. All displayed lanes are contiguous from a single membrane per target; no lanes were spliced, excised, or rearranged. Abbreviations: AKI, acute kidney injury; CHOP, C/EBP-homologous protein; GAPDH, glyceraldehyde-3-phosphate dehydrogenase; GRP78, glucose-regulated protein 78; kDa, kilodalton; MyD88, myeloid differentiation primary response 88; TLR4, Toll-like receptor 4.

A.

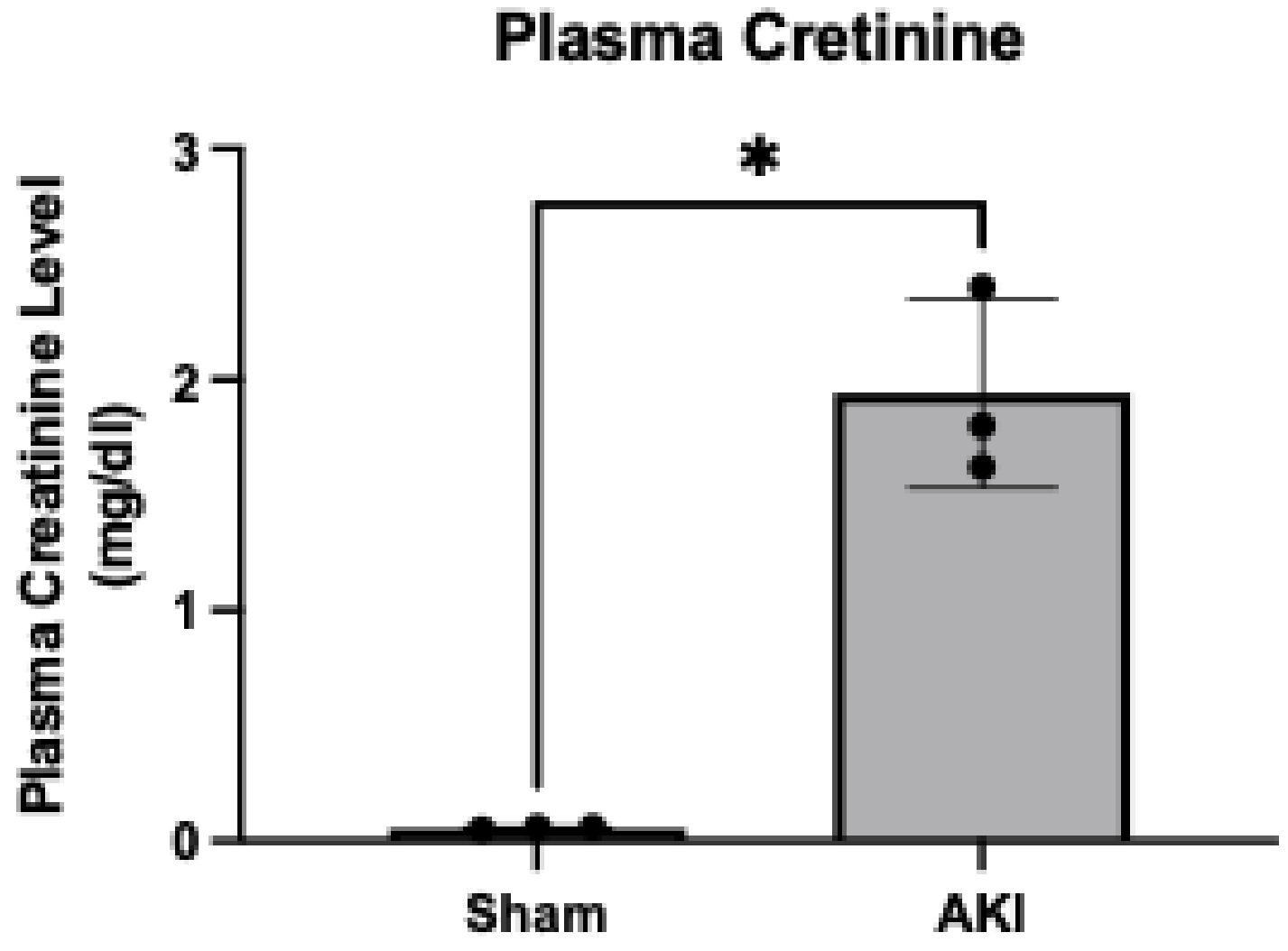
Plasma Cretinine
Plasma Creatinine Level (mg/dl)
3
2
1
0
*
Sham
AKI

B.

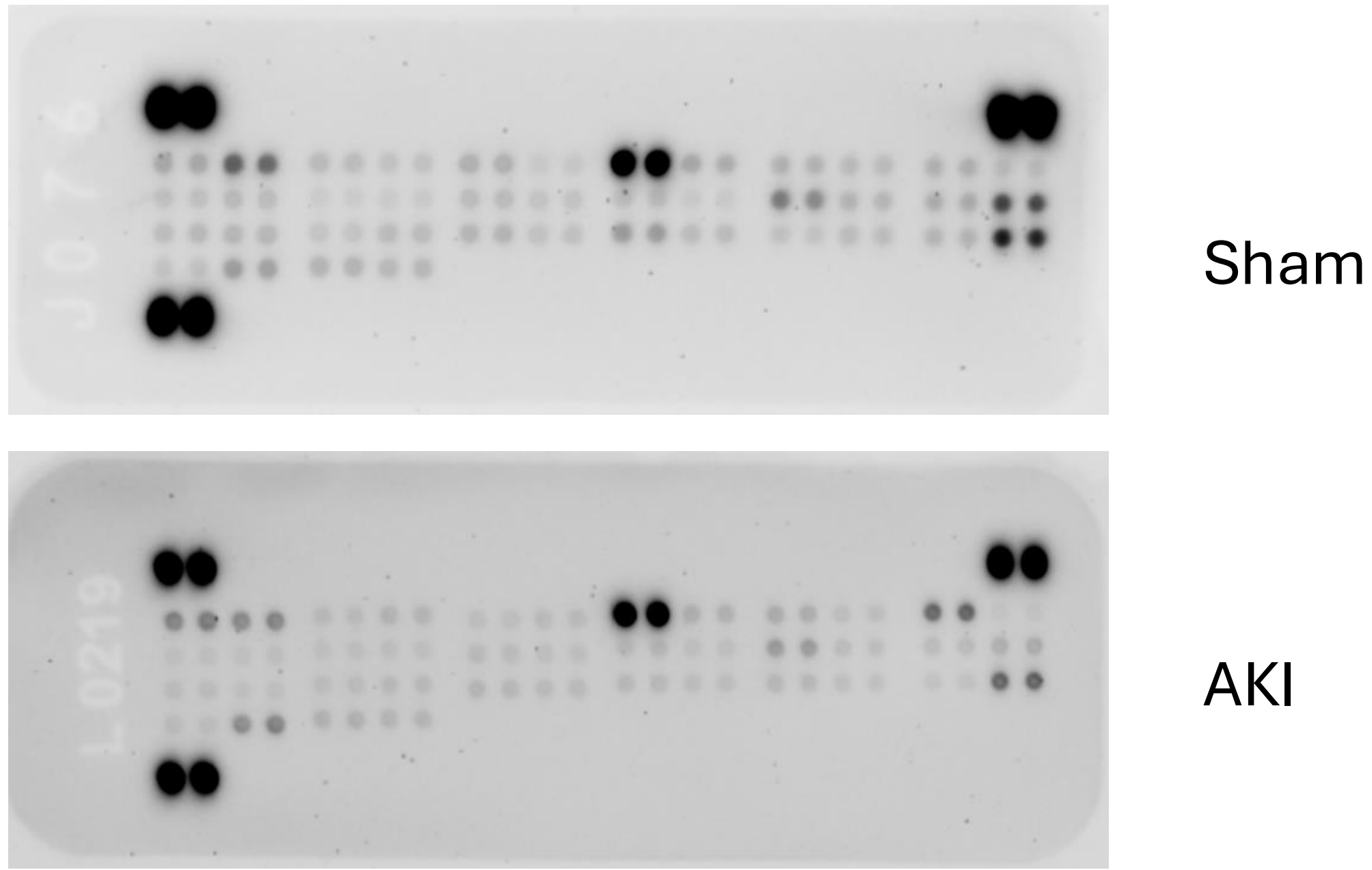
Sham
AKI

C.

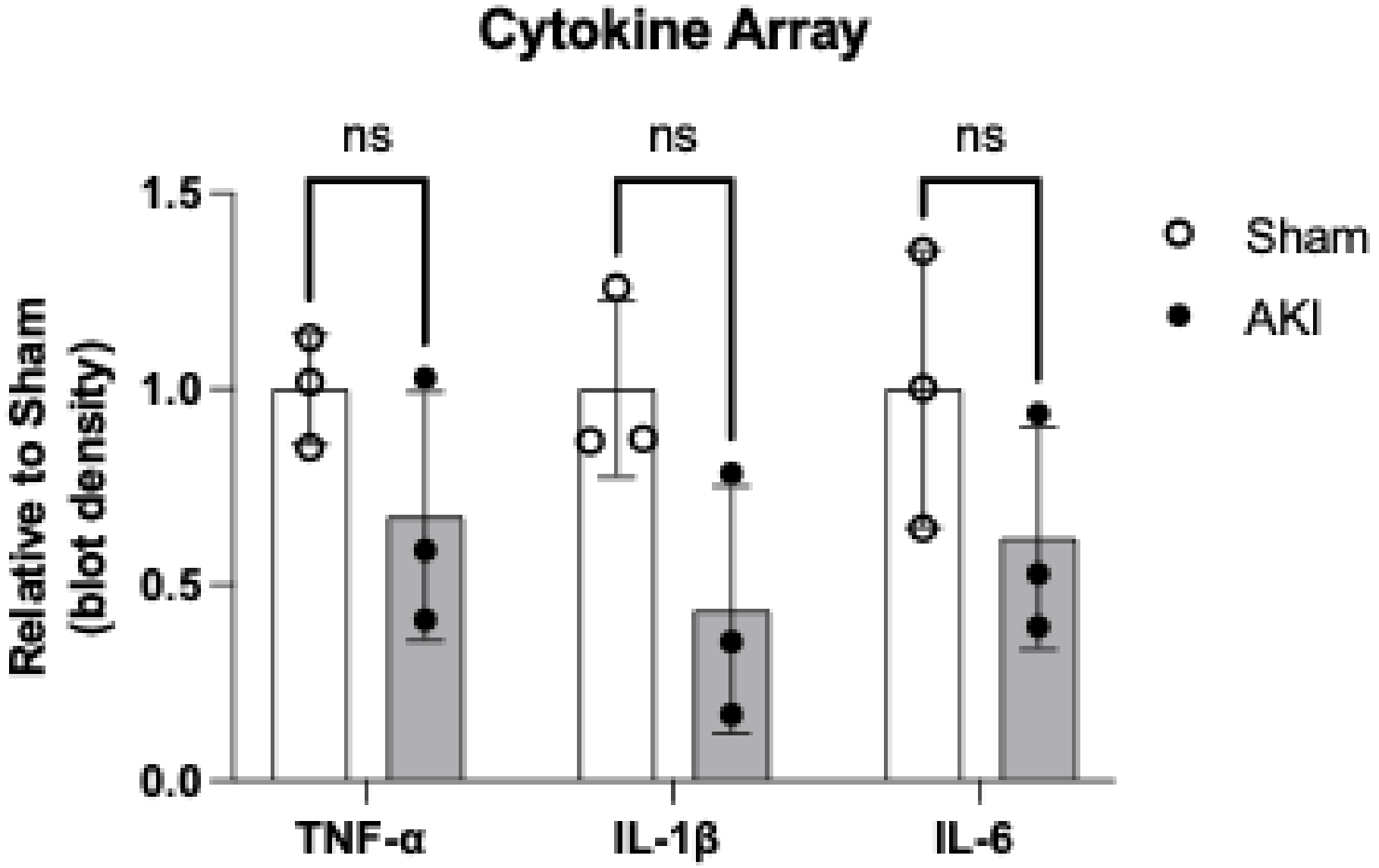
Cytokine Array
Relative to Sham (blot density)
1.5
1.0
0.5
0.0
ns
ns
ns
Sham
AKI
TNF-α
IL-1β
IL-6

S.Fig.1

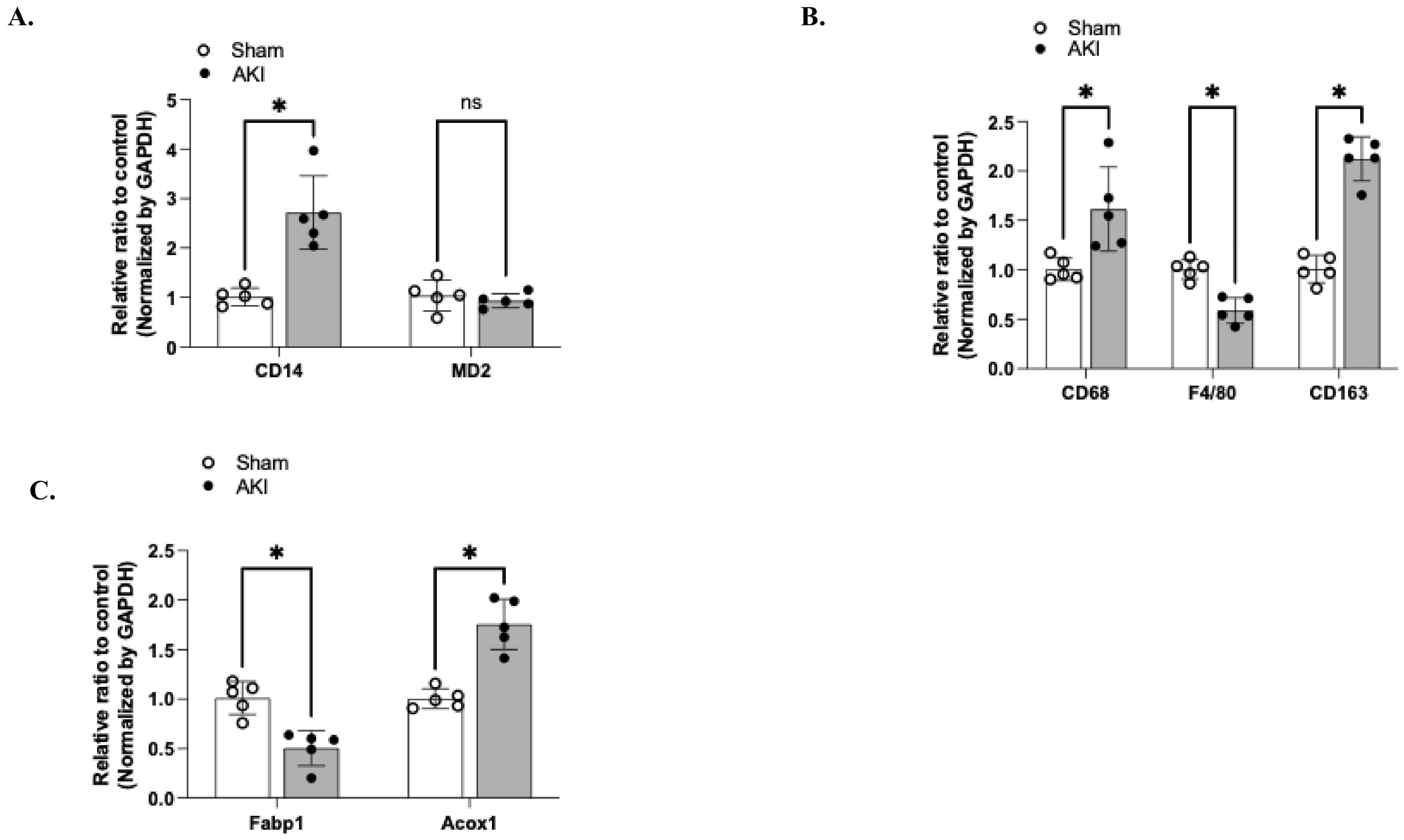

A.
Sham
AKI
Relative ratio to control (Normalized by GAPDH)
*
ns
0
1
2
3
4
5
CD14
MD2
B.
Sham
AKI
Relative ratio to control (Normalized by GAPDH)
*
*
*
0.0
0.5
1.0
1.5
2.0
2.5
CD68
F4/80
CD163
C.
Sham
AKI
Relative ratio to control (Normalized by GAPDH)
*
*
0.0
0.5
1.0
1.5
2.0
2.5
Fabp1
Acox1

S.Fig.2

A.

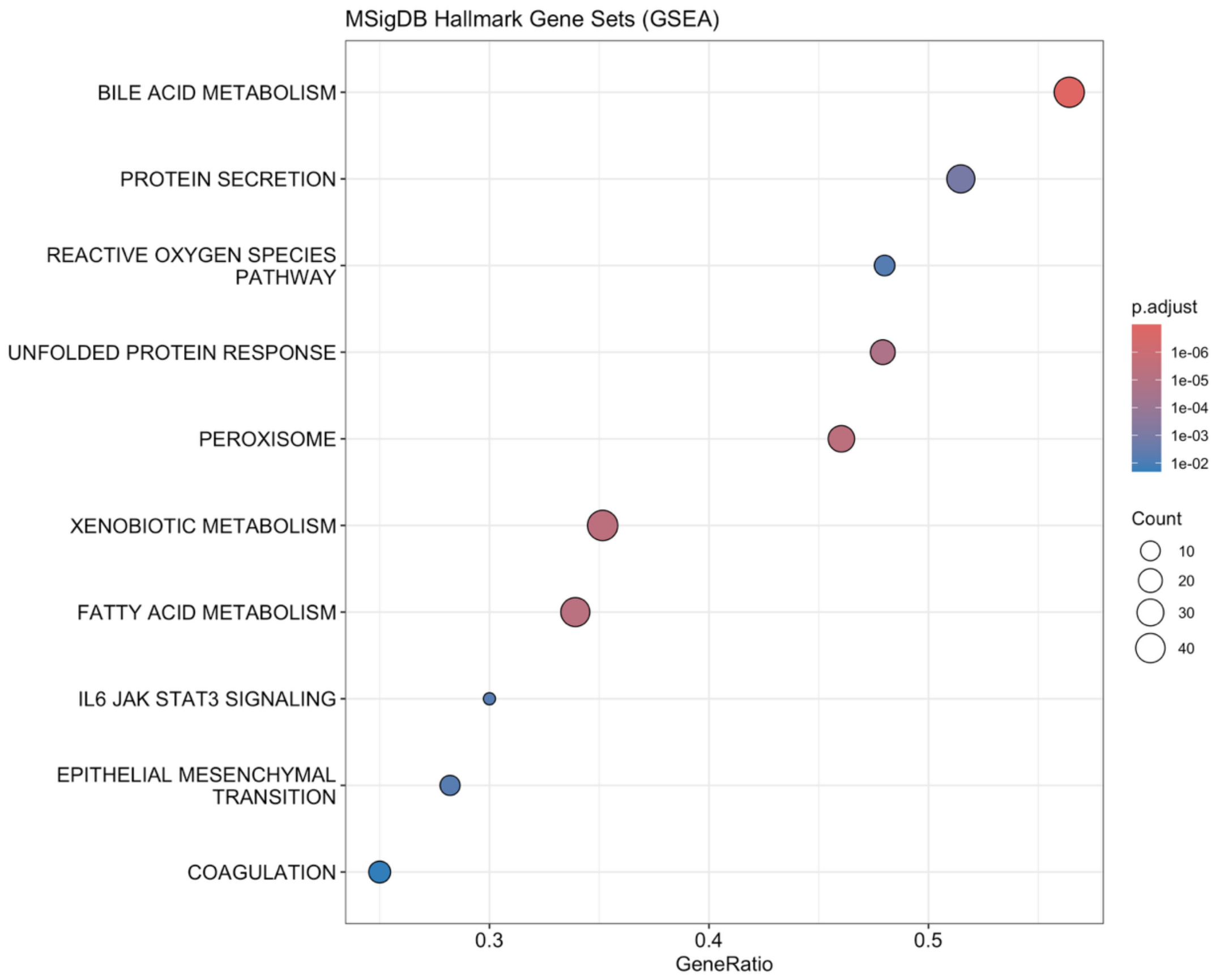
MSigDB Hallmark Gene Sets (GSEA)
BILE ACID METABOLISM
PROTEIN SECRETION
REACTIVE OXYGEN SPECIES PATHWAY
UNFOLDED PROTEIN RESPONSE
PEROXISOME
XENOBIOTIC METABOLISM
FATTY ACID METABOLISM
IL6 JAK STAT3 SIGNALING
EPITHELIAL MESENCHYMAL TRANSITION
COAGULATION
0.3
0.4
0.5
GeneRatio
p.adjust
1e-06
1e-05
1e-04
1e-03
1e-02
Count
10
20
30
40

B.

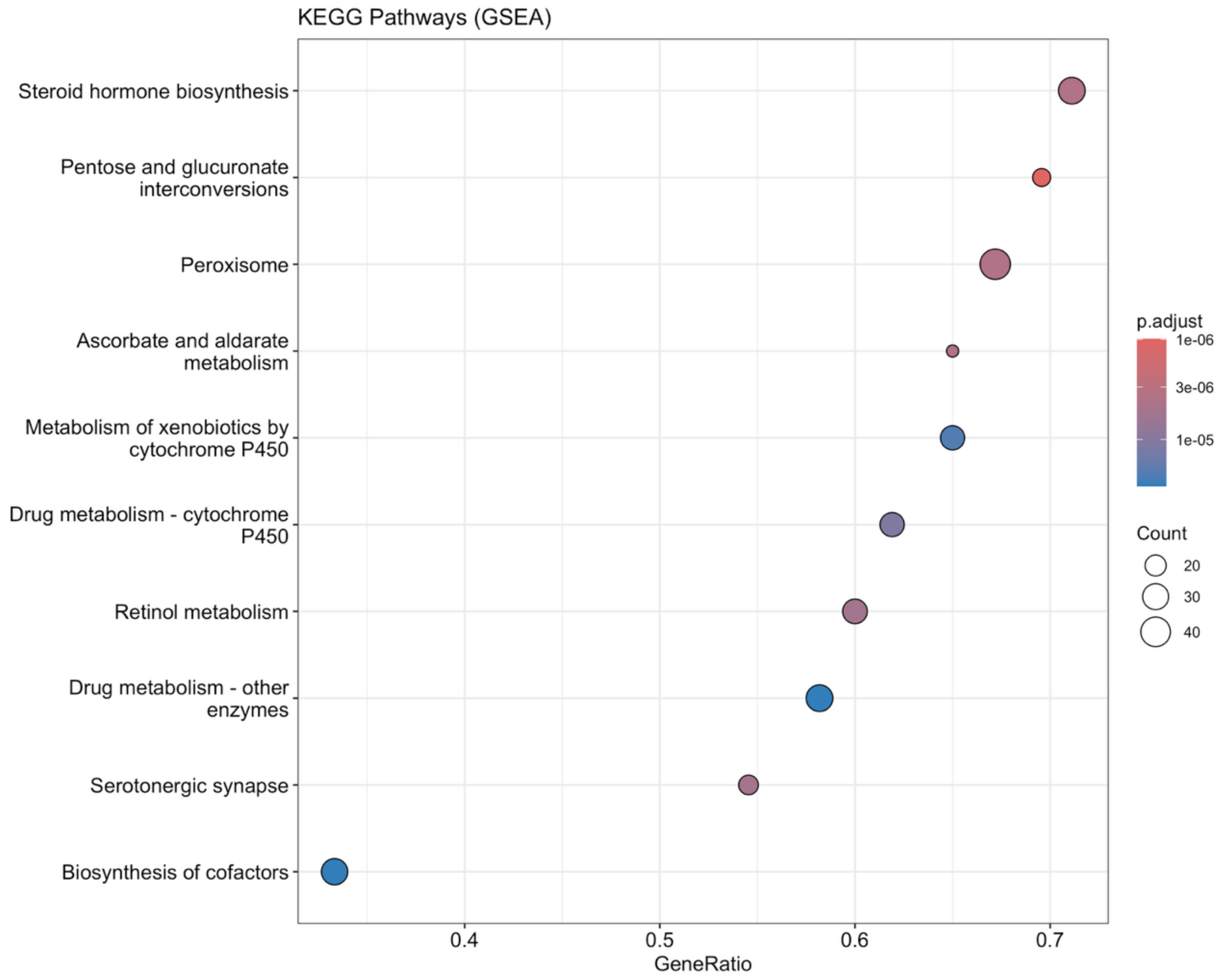
KEGG Pathways (GSEA)
Steroid hormone biosynthesis
Pentose and glucuronate interconversions
Peroxisome
Ascorbate and aldarate metabolism
Metabolism of xenobiotics by cytochrome P450
Drug metabolism - cytochrome P450
Retinol metabolism
Drug metabolism - other enzymes
Serotonergic synapse
Biosynthesis of cofactors
0.4
0.5
0.6
0.7
GeneRatio
p.adjust
1e-06
3e-06
1e-05
Count
20
30
40

S.Fig.3

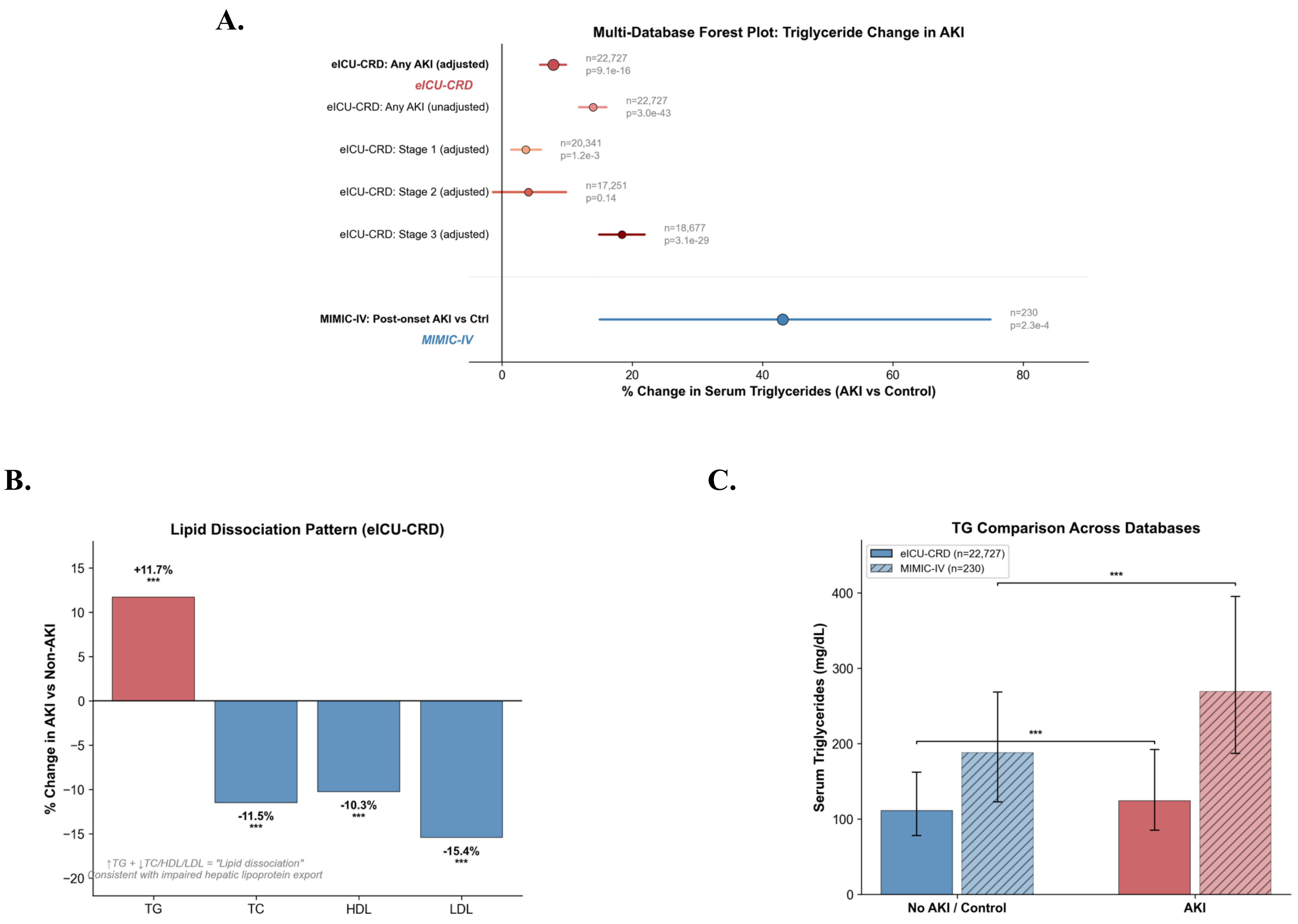

A.
Multi-Database Forest Plot: Triglyceride Change in AKI
eICU-CRD: Any AKI (adjusted)
eICU-CRD
eICU-CRD: Any AKI (unadjusted)
eICU-CRD: Stage 1 (adjusted)
eICU-CRD: Stage 2 (adjusted)
eICU-CRD: Stage 3 (adjusted)
MIMIC-IV: Post-onset AKI vs Ctrl
MIMIC-IV
n=22,727
p=9.1e-16
n=22,727
p=3.0e-43
n=20,341
p=1.2e-3
n=17,251
p=0.14
n=18,677
p=3.1e-29
n=230
p=2.3e-4
0
20
40
60
80
% Change in Serum Triglycerides (AKI vs Control)
B.
Lipid Dissociation Pattern (eICU-CRD)
% Change in AKI vs Non-AKI
+11.7%
-11.5%
-10.3%
-15.4%
***
↑TG + ↓TC/HDL/LDL = "Lipid dissociation"
Consistent with impaired hepatic lipoprotein export
TG
TC
HDL
LDL
C.
TG Comparison Across Databases
eICU-CRD (n=22,727)
MIMIC-IV (n=230)
Serum Triglycerides (mg/dL)
***
No AKI / Control
AKI

S.Fig.4

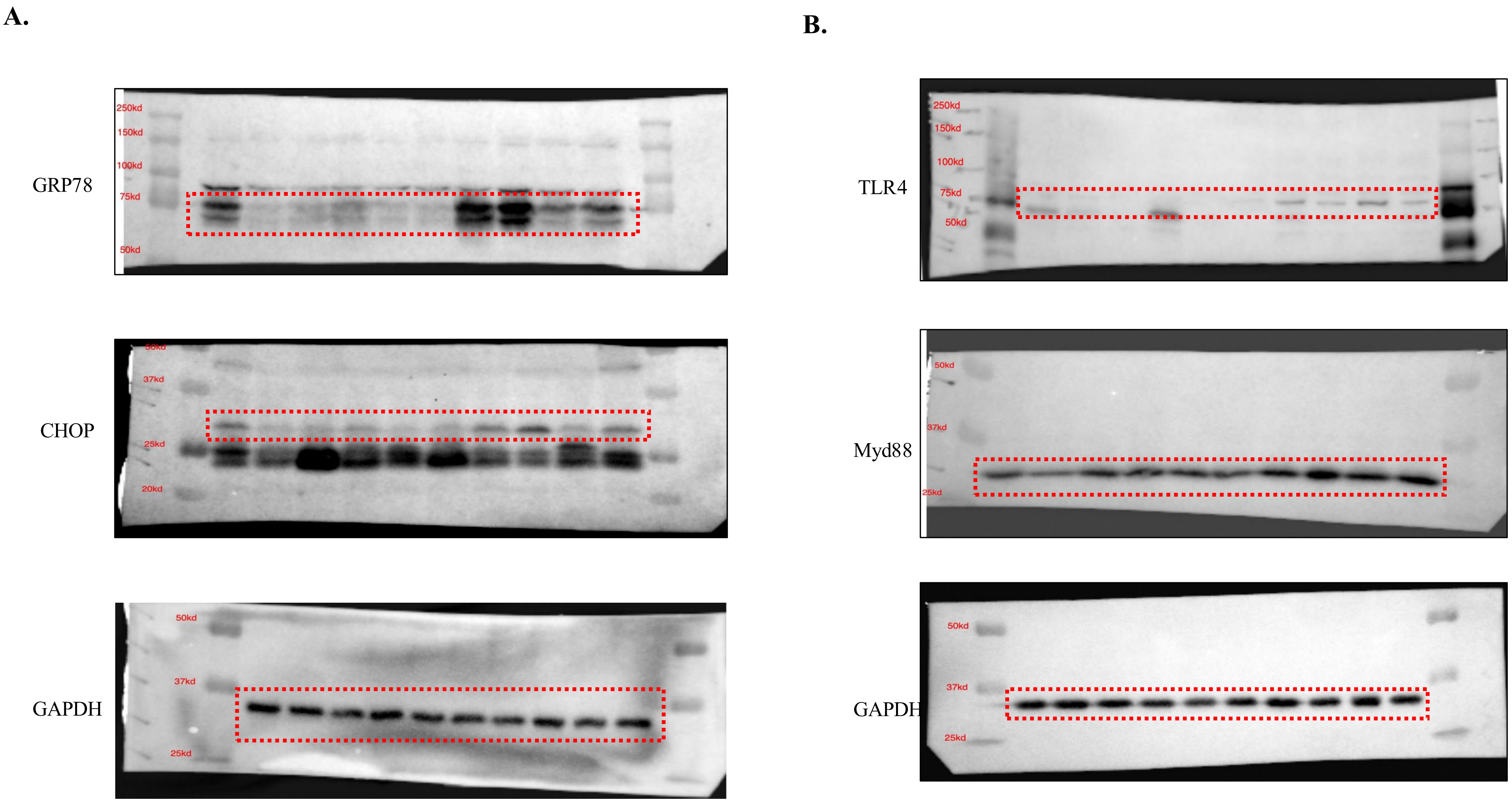
A.
B.
GRP78
250kd
150kd
100kd
75kd
50kd
CHOP
50kd
37kd
25kd
20kd
GAPDH
50kd
37kd
25kd
TLR4
250kd
150kd
100kd
75kd
50kd
Myd88
50kd
37kd
25kd
GAPDH
50kd
37kd
25kd

S.Fig.5

Table S1. Complete list of differentially expressed proteins in liver tissue 24 hours post-AKI

Protein ID	Gene Symbol	Protein Name	log2FC (AKI/Sham)	P-value	Adjusted P-value (FDR)	Mean Intensity Sham	Mean Intensity AKI	Regulation
Q3TF08	*Rbp4*	Retinol-binding protein 4	-3.47	8.58E-11	3.06E-07	24.73	21.26	DOWN
O55239	*Nnmt*	Nicotinamide N-methyltransferase	2.72	1.39E-09	2.22E-06	23.66	26.38	UP
Q91XL1	*Lrg1*	Lrg1 protein	3.99	1.87E-09	2.22E-06	21.43	25.42	UP
E9PV24	*Fga*	Fibrinogen alpha chain	2.54	3.32E-09	2.96E-06	25.52	28.06	UP
Q3UBS3	*Hp*	Haptoglobin	2.45	7.70E-09	5.50E-06	25.81	28.25	UP
Q505A3	*B3galt1*	Hexosyltransferase	2.73	1.12E-08	5.78E-06	19.55	22.28	UP
I3PQW8	*Tifa*	TRAF-interacting protein with FHA domain-containing protein A	4.92	1.13E-08	5.78E-06	18.11	23.03	UP
Q6NWW9	*Fndc3b*	Fibronectin type III domain-containing protein 3B	4.26	1.61E-08	6.92E-06	19.01	23.27	UP
A0A1B0GQV5	*Saa2*	Serum amyloid A protein	10.91	1.75E-08	6.92E-06	17.20	28.10	UP
P01027	*C3*	Complement C3	1.13	2.00E-08	7.12E-06	26.13	27.27	UP
E9PVD2	*Itih4*	Itih4 protein	2.48	3.66E-08	1.16E-05	24.07	26.55	UP
A7E1Z5	*Slc4a4*	Anion exchange protein	1.46	5.95E-08	1.63E-05	22.19	23.65	UP

Protein ID	Gene Symbol	Protein Name	log2FC (AKI/Sham)	P-value	Adjusted P-value (FDR)	Mean Intensity Sham	Mean Intensity AKI	Regulation
P11672	*Lcn2*	Neutrophil gelatinase-associated lipocalin	7.76	6.67E-08	1.70E-05	19.12	26.89	UP
Q8QZR1	*Tat*	Tyrosine aminotransferase	2.40	8.76E-08	1.92E-05	24.05	26.45	UP
Q61838	*A2m*	Pregnancy zone protein	1.28	9.66E-08	1.92E-05	25.73	27.01	UP
Q3UER8	*Fgg*	Fibrinogen gamma chain	2.17	9.68E-08	1.92E-05	26.14	28.30	UP
Q6S9I0	*Kng2*	Kng2 protein	3.41	1.69E-07	3.14E-05	18.90	22.31	UP
Q3TGR2	*Fgb*	Fibrinogen beta chain	2.37	1.94E-07	3.14E-05	25.42	27.78	UP
Q4JFI8	*Apcs*	Pentraxin family member	5.36	2.86E-07	3.68E-05	19.43	24.79	UP
Q3UWC2	*Saa1*	Serum amyloid A protein	7.67	3.07E-07	3.78E-05	20.47	28.13	UP
Q07456	*Ambp*	Protein AMBP	1.07	3.23E-07	3.79E-05	23.46	24.54	UP
Q03734	*Serpina3m*	Serine protease inhibitor A3M	4.26	3.29E-07	3.79E-05	21.34	25.60	UP
Q9CR29	*Ccdc43*	Coiled-coil domain-containing protein 43	2.95	3.95E-07	4.14E-05	19.15	22.10	UP
Q91X72	*Hpx*	Hemopexin	1.73	6.33E-07	6.09E-05	24.89	26.62	UP
Q545K5	*Rbm3*	RNA-binding protein 3	2.29	6.49E-07	6.09E-05	22.91	25.20	UP
P58710	*Gulo*	L-gulonolactone oxidase	-1.07	7.22E-07	6.48E-05	28.67	27.60	DOWN
A0A0R4J0N7	*Cyp8b1*	Cyp8b1 protein	-1.05	7.26E-07	6.48E-05	25.79	24.74	DOWN

Protein ID	Gene Symbol	Protein Name	log2FC (AKI/Sham)	P-value	Adjusted P-value (FDR)	Mean Intensity Sham	Mean Intensity AKI	Regulation
Q3UEK1	*Mbl2*	Mannose-binding protein C	-1.48	7.60E-07	6.61E-05	24.37	22.88	DOWN
P23953	*Ces1c*	Carboxylesterase 1C	-1.25	9.60E-07	7.61E-05	24.67	23.42	DOWN
F2WWK6	*Fads2*	Acyl-CoA 6-desaturase	-1.21	1.35E-06	1.02E-04	24.64	23.43	DOWN
P21981	*Tgm2*	Protein-glutamine gamma-glutamyltransferase 2	1.03	1.79E-06	1.31E-04	26.15	27.18	UP
Q91X83	*Mat1a*	S-adenosylmethionine synthase isoform type-1	1.46	1.90E-06	1.36E-04	29.37	30.83	UP
Q9CQW9	*Ifitm3*	Interferon-induced transmembrane protein 3	1.30	1.99E-06	1.36E-04	24.37	25.67	UP
Q61704	*Itih3*	Inter-alpha-trypsin inhibitor heavy chain H3	4.68	2.05E-06	1.38E-04	18.85	23.53	UP
Q923B6	*Steap4*	Metalloreductase STEAP4	2.73	2.48E-06	1.64E-04	25.67	28.40	UP
Q3U8S0	*Ap3s1*	Ap3s1 protein	3.74	3.34E-06	2.13E-04	19.08	22.82	UP
Q566J8	*Adck4*	Atypical kinase COQ8B, mitochondrial	4.34	3.63E-06	2.23E-04	18.80	23.14	UP
Q99J93	*Ifitm2*	Interferon-induced transmembrane protein 2	2.07	3.98E-06	2.41E-04	22.89	24.97	UP
Q3UKN6	*Nucb2*	Nucb2 protein	3.61	6.69E-06	3.85E-04	20.15	23.76	UP
Q4FJR9	*Isg15*	Ubiquitin-like protein ISG15	-3.12	7.43E-06	4.21E-04	22.63	19.51	DOWN
Q3U5H8	*Hmox1*	Heme oxygenase 1	4.52	1.20E-05	6.17E-04	19.34	23.86	UP

Protein ID	Gene Symbol	Protein Name	log2FC (AKI/Sham)	P-value	Adjusted P-value (FDR)	Mean Intensity Sham	Mean Intensity AKI	Regulation
Q8VCC1	*Hpgd*	15-hydroxyprostaglandin dehydrogenase [NAD(+)]	-3.29	1.69E-05	7.66E-04	23.73	20.43	DOWN
Q9QXT7	*C6*	C6 protein	-2.58	1.84E-05	8.01E-04	24.13	21.55	DOWN
Q3KQQ2	*Mup3*	Mup3 protein	-1.01	1.94E-05	8.15E-04	27.46	26.45	DOWN
A0A0R4J0E1	*Fgl1*	Fibrinogen-like protein 1	5.17	2.23E-05	9.00E-04	19.36	24.54	UP
A2A5E1	*Dnajc7*	DnaJ homolog subfamily C member 7	3.68	2.44E-05	9.24E-04	19.06	22.73	UP
E0CYH9	*Nos1ap*	Nos1ap protein	2.99	2.45E-05	9.24E-04	18.90	21.89	UP
P41233	*Abca1*	Phospholipid-transporting ATPase ABCA1	2.55	3.47E-05	1.17E-03	21.22	23.78	UP
Q5FW60	*Mup20*	Major urinary protein 20	-1.41	3.61E-05	1.20E-03	28.91	27.50	DOWN
E9QAH1	*Golgb1*	Golgb1 protein	1.81	4.13E-05	1.33E-03	21.29	23.10	UP
Q80VJ2	*Sra1*	Steroid receptor RNA activator 1	1.00	5.67E-05	1.67E-03	22.33	23.33	UP
P07309	*Ttr*	Transthyretin	-1.05	5.96E-05	1.73E-03	26.61	25.56	DOWN
Q6P200	*Cyp17a1*	Steroid 17-alpha-hydroxylase/17,20 lyase	2.40	8.12E-05	2.16E-03	19.61	22.01	UP
O70570	*Pigr*	Polymeric immunoglobulin receptor	-2.02	9.14E-05	2.32E-03	24.35	22.33	DOWN
Q80XK1	*Cyp2a5*	Cytochrome P450	-1.39	1.09E-04	2.60E-03	26.37	24.98	DOWN

Protein ID	Gene Symbol	Protein Name	log2FC (AKI/Sham)	P-value	Adjusted P-value (FDR)	Mean Intensity Sham	Mean Intensity AKI	Regulation
Q9BCZ4	*Vimp*	Selenoprotein S	3.51	1.13E-04	2.65E-03	19.89	23.39	UP
Q64726	*Azgp1*	Zinc-alpha-2-glycoprotein	-1.52	1.28E-04	2.89E-03	23.42	21.90	DOWN
Q60590	*Orm1*	Alpha-1-acid glycoprotein 1	2.44	1.33E-04	2.95E-03	23.27	25.71	UP
A1L3S6	*Cyp7a1*	Cholesterol 7-alpha-monooxygenase	-2.70	1.45E-04	3.11E-03	24.05	21.35	DOWN
G3X8T9	*Serpina3n*	Serpina3n protein	5.06	1.52E-04	3.21E-03	20.54	25.61	UP
F2Z3U3	*Raph1*	Raph1 protein	1.32	1.58E-04	3.29E-03	22.25	23.57	UP
A0A077S2U6	*Lyz2*	lysozyme	3.78	1.87E-04	3.66E-03	19.79	23.56	UP
Q9D566	*Sult1e1*	Sulfotransferase	4.19	2.13E-04	4.02E-03	18.70	22.90	UP
P02802	*Mt1*	Metallothionein-1	6.01	2.62E-04	4.58E-03	23.42	29.42	UP
Q3TDW9	*Ralbp1*	Ralbp1 protein	-1.51	2.65E-04	4.58E-03	20.30	18.79	DOWN
A0A0N4SVU8	*Tmem176a*	Tmem176a protein	2.58	2.92E-04	4.90E-03	19.22	21.79	UP
Q3U711	*Plin2*	Perilipin	2.00	3.54E-04	5.59E-03	24.03	26.02	UP
P60824	*Cirbp*	Cold-inducible RNA-binding protein	2.69	3.99E-04	6.10E-03	19.18	21.87	UP
A0A0A6YW87	*Cnih4*	Cnih4 protein	-3.59	4.14E-04	6.26E-03	22.56	18.96	DOWN
Q4FK16	*Plscr1*	Phospholipid scramblase	1.04	4.30E-04	6.44E-03	22.69	23.73	UP

Protein ID	Gene Symbol	Protein Name	log2FC (AKI/Sham)	P-value	Adjusted P-value (FDR)	Mean Intensity Sham	Mean Intensity AKI	Regulation
Q75N73	*Slc39a14*	Metal cation symporter ZIP14	1.59	4.35E-04	6.48E-03	23.62	25.22	UP
Q8VC77	*Akr1c20*	Akr1c20 protein	-1.23	4.84E-04	6.99E-03	24.05	22.82	DOWN
A2CEK7	*Mup14*	Mup14 protein	-1.22	6.67E-04	8.94E-03	31.68	30.46	DOWN
A2AKN8	*Mup8*	Mup8 protein	-1.32	8.02E-04	1.02E-02	28.34	27.02	DOWN
Q9CY29	*Psmd4*	26S proteasome non-ATPase regulatory subunit 4	2.62	8.14E-04	1.03E-02	19.12	21.73	UP
A0A1Y7VK19	*Isca2*	Iron-sulfur cluster assembly 2 homolog, mitochondrial	2.70	8.85E-04	1.10E-02	19.29	21.99	UP
G3UZD6	*Ube4b*	Ubiquitin conjugation factor E4	1.07	1.16E-03	1.36E-02	19.67	20.75	UP
Q9EQ21	*Hamp*	Hepcidin	2.13	1.16E-03	1.36E-02	21.69	23.83	UP
B2ZRS3	*Deptor*	DEP domain-containing mTOR-interacting protein	-1.50	1.28E-03	1.44E-02	23.53	22.03	DOWN
Q8K2H5	*Maged1*	Melanoma-associated antigen D1	2.09	1.28E-03	1.44E-02	19.67	21.76	UP
Q61408	*Gm4788*	Gm4788 protein	-2.33	1.35E-03	1.49E-02	21.84	19.50	DOWN
Q3UHE7	*Kif21a*	Kinesin motor domain-containing protein	1.04	1.36E-03	1.49E-02	23.81	24.84	UP
Q9QYE6	*Golga5*	Golgin subfamily A member 5	1.56	1.41E-03	1.52E-02	20.73	22.29	UP

Protein ID	Gene Symbol	Protein Name	log2FC (AKI/Sham)	P-value	Adjusted P-value (FDR)	Mean Intensity Sham	Mean Intensity AKI	Regulation
A0A494BBF6	*Stub1*	E3 ubiquitin-protein ligase CHIP	1.14	1.50E-03	1.59E-02	19.93	21.08	UP
Q544T4	*St3gal1*	CMP-N-acetylneuraminate-beta-galactosamide-alpha-2,3-sialyltransferase 1	2.53	1.56E-03	1.65E-02	20.24	22.77	UP
A0A0R4J1J3	*St3gal5*	Lactosylceramide alpha-2,3-sialyltransferase	1.99	1.59E-03	1.67E-02	22.89	24.88	UP
A2BIN1	*Mup10*	Mup10 protein	-1.66	1.61E-03	1.67E-02	29.64	27.99	DOWN
Q9CR58	*Slc25a30*	Kidney mitochondrial carrier protein 1	2.38	1.66E-03	1.70E-02	19.56	21.94	UP
Q9CX98	*Cyp2u1*	Cytochrome P450 2U1	-3.22	1.73E-03	1.74E-02	22.09	18.88	DOWN
Q99JZ7	*Errfi1*	ERBB receptor feedback inhibitor 1	1.57	1.74E-03	1.74E-02	21.93	23.50	UP
Q8BIL5	*Hook1*	Protein Hook homolog 1	1.00	1.79E-03	1.77E-02	21.31	22.31	UP
Q9CXK3	*Actc1*	ACTC protein	-1.12	1.99E-03	1.92E-02	23.64	22.52	DOWN
F8VQ05	*Fryl*	Fryl protein	2.67	2.06E-03	1.96E-02	19.87	22.55	UP
Q8VCT0	*Ldlr*	Low-density lipoprotein receptor	-2.47	2.06E-03	1.96E-02	23.05	20.58	DOWN
A8CA96	*Tcea3*	Transcription elongation factor	-2.23	2.18E-03	2.05E-02	20.86	18.63	DOWN
E9Q1P8	*Irf2bp2*	Interferon regulatory factor 2-binding protein 2	1.55	2.26E-03	2.10E-02	19.62	21.17	UP
P28651	*Ca8*	Carbonic anhydrase-related protein	-1.71	2.29E-03	2.12E-02	22.77	21.06	DOWN

Protein ID	Gene Symbol	Protein Name	log2FC (AKI/Sham)	P-value	Adjusted P-value (FDR)	Mean Intensity Sham	Mean Intensity AKI	Regulation
Q99LC9	*Pex6*	Peroxisomal ATPase PEX6	-1.08	2.50E-03	2.23E-02	23.27	22.20	DOWN
Q9R1Q6	*Tmem176b*	Transmembrane protein 176B	1.48	2.65E-03	2.30E-02	20.06	21.54	UP
Q9CTW2	*Lipe*	Hormone-sensitive lipase	-1.48	2.69E-03	2.31E-02	21.01	19.53	DOWN
O08692	*Ngp*	Neutrophilic granule protein	1.25	2.69E-03	2.31E-02	22.58	23.83	UP
A0A0R4J0T8	*Arfgap3*	ADP-ribosylation factor GTPase-activating protein 3	2.31	2.72E-03	2.32E-02	19.78	22.09	UP
Q9EQQ9	*Mgea5*	Protein O-GlcNAcase	1.36	3.05E-03	2.53E-02	19.51	20.87	UP
Q8CC91	*Cyp2j6*	unspecific monooxygenase	-1.84	3.08E-03	2.54E-02	21.82	19.98	DOWN
Q3TUU3	*Acaa1b*	Acaa1b protein	-1.22	3.13E-03	2.57E-02	28.67	27.46	DOWN
B7ZMW6	*Cd163*	Scavenger receptor cysteine-rich type 1 protein M130	2.69	3.35E-03	2.68E-02	20.62	23.31	UP
Q3UGS4	*Fam195b*	Mapk-regulated corepressor-interacting protein 1	2.18	3.64E-03	2.86E-02	19.22	21.40	UP
A0A097BVN3	*Gfra1*	GDNF family receptor alpha	1.40	3.73E-03	2.92E-02	21.11	22.51	UP
Q3UN35	*B4galnt1*	Beta-1,4 N-acetylgalactosaminyltransferase	2.42	3.81E-03	2.97E-02	20.04	22.45	UP
Q547C4	*Scd1*	stearoyl-CoA 9-desaturase	-1.59	4.05E-03	3.08E-02	25.46	23.87	DOWN

Protein ID	Gene Symbol	Protein Name	log2FC (AKI/Sham)	P-value	Adjusted P-value (FDR)	Mean Intensity Sham	Mean Intensity AKI	Regulation
Q9D1K2	*Atp6v1f*	V-type proton ATPase subunit F	-2.60	4.08E-03	3.10E-02	21.96	19.37	DOWN
Q8VE91	*Fam134b*	Reticulophagy regulator 1	2.06	4.12E-03	3.10E-02	19.34	21.40	UP
Q8C094	*Mapk9*	Stress-activated protein kinase JNK	-2.19	4.28E-03	3.17E-02	23.04	20.85	DOWN
Q80Y55	*Bsdc1*	BSD domain-containing protein 1	1.14	4.30E-03	3.17E-02	20.62	21.75	UP
Q03311	*Bche*	Cholinesterase	-1.90	4.44E-03	3.24E-02	23.54	21.65	DOWN
Q4FJZ2	*Kpna6*	Importin subunit alpha	-1.11	4.74E-03	3.39E-02	23.16	22.05	DOWN
B7ZWM6	*Sorbs2*	Sorbs2 protein	-2.48	4.84E-03	3.43E-02	21.77	19.30	DOWN
B2RWZ2	*Myo1d*	Unconventional myosin-Id	2.19	4.93E-03	3.46E-02	19.35	21.54	UP
A0A140T8I9	*Pi4ka*	Phosphatidylinositol 4-kinase alpha	1.10	5.40E-03	3.69E-02	19.76	20.86	UP
A0A494BBG2	*Fam195a*	Fam195a protein	-2.47	5.61E-03	3.76E-02	22.05	19.58	DOWN
Q14BE1	*Slc35d1*	Slc35d1 protein	-1.56	6.27E-03	4.09E-02	25.09	23.52	DOWN
A0A571BEC9	*Plin4*	Perilipin-4	2.98	6.38E-03	4.14E-02	18.72	21.70	UP
Q8R2Q8	*Bst2*	Bone marrow stromal antigen 2	-1.76	6.51E-03	4.15E-02	23.05	21.29	DOWN
Q3UVH2	*Csk*	Tyrosine-protein kinase	-1.02	6.56E-03	4.17E-02	23.49	22.47	DOWN
E9QA15	*Cald1*	Cald1 protein	3.23	6.83E-03	4.23E-02	18.53	21.77	UP

Protein ID	Gene Symbol	Protein Name	log2FC (AKI/Sham)	P-value	Adjusted P-value (FDR)	Mean Intensity Sham	Mean Intensity AKI	Regulation
Q8VD00	*Tmem97*	Sigma intracellular receptor 2	-1.74	7.47E-03	4.48E-02	25.02	23.28	DOWN
O35728	*Cyp4a14*	Cytochrome P450 4A14	3.76	7.62E-03	4.56E-02	20.28	24.04	UP
Q3UPN9	*Cebpb*	CCAAT/enhancer-binding protein	2.48	7.68E-03	4.58E-02	19.43	21.91	UP
P06728	*Apoa4*	Apolipoprotein A-IV	1.43	7.84E-03	4.64E-02	21.82	23.25	UP

FC, fold change; FDR, false discovery rate. Significance threshold: $|log2FC| > 0.25$, $FDR < 0.05$

Table S2. Gene Set Enrichment Analysis (GSEA) of hepatic proteome following AKI

Pathway Name	Database	Gene Set Size	NES	P-value	FDR q-value	Leading Edge Genes
**A. Significantly UPREGULATED pathways (positive NES, FDR < 0.25)**						
Network Map Of SARS-CoV-2 Signaling	WikiPathways	73	**2.10**	3.39e-07	3.90e-05	*Lrg1, Fga, Hp, Saa2, Itih4, Ctsl, Fgg, Fgb*
Unfolded Protein Response †	Hallmark	48	**2.08**	1.95e-06	1.27e-05	*Fus, Eef2, Arfgap1, Khsrp, Srprb, Eif4a3, Bag3, Dnajc3*
Cytoplasmic Ribosomal Proteins	WikiPathways	74	**2.05**	5.47e-07	3.90e-05	*Rpl23a, Rpl18a, Rpl13a, Rps9, Rpl28, Rps27, Rps3a1, Rpl18*
Spliceosome	KEGG	82	**2.00**	5.09e-06	9.51e-05	*Ddx5, Fus, Puf60, Snrpf, Eif4a3, Sart1, Rbm8a, Sf3a1*
Vitamin B12 Metabolism	WikiPathways	29	**1.97**	9.91e-05	2.84e-03	*Fga, Saa2, Fgg, Fgb, Saa1, Serpina3m, Mat1a, Abca1*
Integrin Signaling	KEGG	42	**1.93**	1.74e-04	2.12e-03	*Fga, C3, Fgg, Fgb, Fn1, Vtn, Fermt2*
mRNA Processing	WikiPathways	77	**1.92**	1.57e-05	6.39e-04	*Fus, Hnrnpl, Snrpf, Sfpq, Sf3a1, Srsf5, Ppm1g, Sf3b1*
IL6 JAK Stat3 Signaling	Hallmark	10	**1.85**	1.29e-03	4.69e-03	*A2m, Stat3, Hmox1*
Complement And Coagulation Cascades	KEGG	34	**1.84**	9.94e-04	1.01e-02	*Fga, C3, A2m, Fgg, Kng2, Fgb, Kng1, Clu, Vtn, Serpinf2, Serpina1b*
Protein Secretion †	Hallmark	68	**1.83**	1.56e-04	8.47e-04	*Ap3s1, Sec24d, Abca1, Gbf1, Sec22b, Tpd52, Arfgap3, Uso1*
Epithelial Mesenchymal Transition †	Hallmark	39	**1.78**	9.39e-04	4.38e-03	*Nnmt, Fn1, Tgm2, Cald1, Lrp1, Fermt2, Calu, Tpm1*
Translation Factors	WikiPathways	40	**1.77**	9.18e-04	1.64e-02	*Eif6, Eef2, Pabpc1, Eif3g, Eif4g1, Eif3b, Eif4a1, Eif4h*
Blood Clotting Cascade	WikiPathways	10	**1.74**	4.53e-03	4.61e-02	*Fga, Fgg, Fgb, Serpinf2*
Folate Metabolism	WikiPathways	35	**1.74**	2.71e-03	3.87e-02	*Fga, Saa2, Fgg, Fgb, Saa1, Serpina3m, Mat1a, Abca1*
Neutrophil Extracellular Trap Formation	KEGG	29	**1.72**	3.93e-03	2.99e-02	*Fga, C3, Fgg, Fgb*
MirTargeted Genes In Squamous Cell	WikiPathways	61	**1.72**	1.95e-03	3.27e-02	*Fndc3b, Hmox1, Csde1, Atp2a2, Gfpt1, Ptma, Srprb, Akap8*
Ribosome	KEGG	138	**1.71**	6.20e-05	8.38e-04	*Mrps5, Rpl23a, Mrps7, Mrps33, Rpl18a, Rpl13a, Rps9, Rpl28*
Affected Pathways In Duchenne Muscular Dystrophy	WikiPathways	19	**1.71**	7.56e-03	6.58e-02	*Fga, Fgg, Fgb*

Pathway Name	Database	Gene Set Size	NES	P-value	FDR q-value	Leading Edge Genes
Burn Wound Healing	WikiPathways	16	**1.65**	8.13e-03	6.70e-02	*Ambp, Fn1, Tpt1, S100a9*
Cytoskeleton In Muscle Cells	KEGG	74	**1.64**	2.72e-03	2.28e-02	*Fn1, Sptbn1, Zyx, Plec, Myh9, Lmna, Sptan1, Tpm1*
Complement System	WikiPathways	33	**1.63**	1.09e-02	8.15e-02	*Fga, C3, Fgg, Fgb, Apcs, Vtn*
Coagulation	Hallmark	60	**1.59**	4.65e-03	1.52e-02	*Fga, C3, Ctsl, A2m, Fgg, Fn1, Clu, Lrp1*
Hypoxia	Hallmark	51	**1.57**	5.92e-03	1.75e-02	*Cp, Tgm2, Pck1, Hmox1, Hdlbp, Mt1, Aldoa, Plin2*
Proteoglycans In Cancer	KEGG	58	**1.55**	1.08e-02	6.94e-02	*Ctsl, Fn1, Stat3, Ddx5, Vtn, Ppp1r12a, Cttn, Actb*
G2/M Checkpoint	Hallmark	49	**1.52**	1.42e-02	3.86e-02	*Sfpq, Rbm14, Kif5b, Srsf1, Numa1, Chmp1a, Syncrip, Ncl*
MirTargeted Genes In Muscle Cell	WikiPathways	133	**1.51**	3.90e-03	4.61e-02	*Fndc3b, Hmox1, Csde1, Atp2a2, Gfpt1, Ptma, Srprb, Lrp1*
Coronavirus Disease - COVID-19	KEGG	104	**1.46**	9.19e-03	6.20e-02	*Fga, C3, Fgg, Fgb, Stat3, Rpl23a, Rpl18a, Rpl13a*
**B. Significantly DOWNREGULATED pathways (negative NES, FDR < 0.25)**						
Pentose And Glucuronate Interconversions	KEGG	23	**-2.36**	3.45e-09	8.39e-07	*Dcxr, Fggy, Ugt1a1, Xylb, Akr1a1, Sord, Ugt1a6b, Dhdh*
Disorders Of Bile Acid Synthesis And Biliary Transport †	WikiPathways	21	**-2.33**	5.97e-08	1.70e-05	*Akr1d1, Baat, Slc10a1, Abcb4, Abcb11, Amacr, Hsd3b7, Scp2*
Steroid Hormone Biosynthesis †	KEGG	45	**-2.28**	4.62e-08	3.35e-06	*Hsd17b6, Hsd11b1, Srd5a1, Akr1d1, Cyp2c70, Hsd3b5, Cyp3a25, Cyp2d22*
Bile Acid Metabolism †	Hallmark	78	**-2.28**	2.30e-09	7.51e-08	*Slc22a18, Slc27a5, Hacl1, Agxt, Sod1, Gnmt, Hsd17b6, Dio1*
Serotonergic Synapse	KEGG	33	**-2.28**	9.82e-08	4.50e-06	*Itpr2, Mapk3, Cyp2c70, Prkacb, Cyp2d22, Cyp2c54, Cyp2c67, Cyp2c68*
Peroxisome †	KEGG	64	**-2.26**	5.06e-08	3.35e-06	*Pex11b, Nudt12, Pex7, Pex26, Acnat1, Dhrs4, Pex3, Hacl1*
Retinol Metabolism	KEGG	45	**-2.25**	1.11e-07	4.50e-06	*Hsd17b6, Rdh16, Cyp2c70, Adh1, Cyp3a25, Cyp2c54, Cyp2c67, Ugt1a1*
Ascorbate And Aldarate Metabolism	KEGG	20	**-2.24**	5.52e-08	3.35e-06	*Rgn, Ugt1a1, Akr1a1, Ugt1a6b, Aldh3a2, Ugt2b35, Ugt2a3, Ugt2b1*
Drug Metabolism - Cytochrome P450	KEGG	42	**-2.23**	2.75e-07	9.56e-06	*Ugt1a9, Gsto1, Adh4, Gstm2, Gstt1, Gstm7, Gstt3, Gstm3*
Metabolism Of Xenobiotics By Cytochrome P450	KEGG	40	**-2.21**	6.16e-07	1.87e-05	*Ugt1a9, Gsto1, Adh4, Gstm2, Gstt1, Gstm7, Hsd11b1, Gstt3*
Oxysterols Derived From Cholesterol	WikiPathways	27	**-2.20**	3.11e-06	1.48e-04	*Hsd11b1, Akr1d1, Baat, Acot8, Amacr, Hsd3b7, Acot12, Scp2*

Pathway Name	Database	Gene Set Size	NES	P-value	FDR q-value	Leading Edge Genes
Peroxisome †	Hallmark	63	**-2.19**	2.63e-07	3.56e-06	*Cadm1, Idh1, Acot8, Abcb4, Pex14, Acsl5, Ehhadh, Hsd3b7*
Chemical Carcinogenesis - DNA Adducts	KEGG	47	**-2.17**	1.44e-06	3.18e-05	*Gstm2, Gstt1, Gstm7, Hsd11b1, Nat2, Gstt3, Gstm3, Mgst1*
Drug Metabolism - Other Enzymes	KEGG	55	**-2.16**	9.16e-07	2.47e-05	*Ces1e, Ugt1a9, Gsto1, Tpmt, Nme3, Gstm2, Gstt1, Gstm7*
Bile Secretion †	KEGG	37	**-2.14**	3.27e-06	6.62e-05	*Baat, Prkacb, Slc10a1, Abcb4, Abcb11, Ugt1a1, Ugt1a6b, Ldlr*
Metapathway Biotransformation Phase I And II	WikiPathways	72	**-2.12**	4.89e-07	3.90e-05	*Mgst1, Gstz1, Akr1d1, Baat, Cyp4v3, Cyp3a25, Cyp2d22, Gsta3*
Oxidation By Cytochrome P450	WikiPathways	24	**-2.06**	6.59e-05	2.35e-03	*Cyp4v3, Cyp3a25, Cyp2d22, Cyp4f15, Cyp27a1, Cyb5r3, Cyp2u1, Cyb5a*
Arachidonic Acid Metabolism	KEGG	28	**-2.06**	3.37e-05	5.46e-04	*Ptgr2, Cyp2c70, Cyp2c54, Cyp2c67, Cyp2c68, Cyp4a12a, Cyp2c37, Cyp2j6*
Glucuronidation	WikiPathways	13	**-2.05**	9.96e-05	2.84e-03	*Ugt1a1, Ugt1a6b, Ugp2, Ugt2b35, Ugt2a3, Ugt2b1, Ugt2b36, Ugt2b34*
Biosynthesis Of Cofactors	KEGG	93	**-2.04**	1.02e-06	2.48e-05	*Lipt1, Coasy, Gphn, Pnpo, Rgn, Gclm, Spr, Ugt1a1*
Biosynthesis Of Unsaturated Fatty Acids	KEGG	23	**-2.04**	4.62e-05	7.02e-04	*Elovl6, Hacd3, Baat, Elovl2, Acox3, Acaa1a, Scp2, Scd1*
ADHD And Autism ASD Pathways	WikiPathways	100	**-2.03**	7.03e-07	4.01e-05	*Sod1, Gnmt, Akt2, Pdxk, Ahcy, Prkacb, Agmo, Gphn*
Fatty Acid Metabolism †	KEGG	46	**-2.03**	3.12e-05	5.42e-04	*Acads, Ppt2, Elovl6, Fasn, Hacd3, Acadsb, Acsl5, Ehhadh*
Tryptophan Metabolism	KEGG	33	**-2.02**	5.31e-05	7.60e-04	*Aldh9a1, Afmid, Dhtkd1, Maob, Hadha, Dld, Dlst, Aldh7a1*
Fatty Acid Metabolism †	Hallmark	115	**-2.02**	5.35e-07	4.36e-06	*Hibch, Fasn, Gcdh, Sucla2, Eci1, Gstz1, Mcee, Adh1*
Xenobiotic Metabolism †	Hallmark	128	**-2.01**	3.27e-07	3.56e-06	*Adh1, Idh1, Dhrs1, Apoe, Gsta3, Dcxr, Fah, Cndp2*
7-OxoC And 7-BetaHC Pathways	WikiPathways	21	**-1.96**	4.45e-04	1.02e-02	*Slc27a5, Hsd11b1, Baat, Acot8, Amacr, Acot12, Scp2, Cyp27a1*
Omega-6 Fatty Acids In Senescence	WikiPathways	21	**-1.95**	4.64e-04	1.02e-02	*Cbr1, Gstp1, Ptgs1, Lta4h, Ptges2, Gpx1, Ptgr2, Cyp4f15*
Codeine And Morphine Metabolism	WikiPathways	11	**-1.95**	3.30e-04	8.55e-03	*Cyp2d22, Ugt1a1, Ugt1a6b, Ugt2b35, Ugt2b1, Ugt2b36, Ugt2b34, Slco1b2*
Primary Bile Acid Biosynthesis †	KEGG	14	**-1.95**	1.08e-04	1.38e-03	*Acnat1, Slc27a5, Akr1d1, Baat, Acot8, Amacr, Hsd3b7, Scp2*
Omega-3 Fatty Acids In Senescence	WikiPathways	15	**-1.92**	8.71e-04	1.64e-02	*Gstp1, Ptgs1, Cyp3a11, Lta4h, Cyp2d22, Elovl2, Ephx2, Fads1*
Disorders Of Folate Metabolism And Transport	WikiPathways	10	**-1.92**	5.14e-04	1.05e-02	*Aldh1l1, Shmt1, Qdpr, Shmt2, Ftcd, Mthfd1*

Pathway Name	Database	Gene Set Size	NES	P-value	FDR q-value	Leading Edge Genes
Valine, Leucine And Isoleucine Degradation	KEGG	44	**-1.90**	2.17e-04	2.52e-03	*Dbt, Aldh9a1, Agxt2, Hadha, Dld, Hsd17b10, Mccc2, Aldh7a1*
Folate Transport And Metabolism	KEGG	12	**-1.89**	8.46e-04	8.94e-03	*Slco2b1, Shmt1, Shmt2, Abcg2, Mthfd1, Slco1b2*
Glyoxylate And Dicarboxylate Metabolism	KEGG	27	**-1.84**	1.15e-03	1.12e-02	*Hao1, Cs, Afmid, Agxt2, Gldc, Dld, Agxt, Glyctk*
Glycogen Synthesis And Degradation	WikiPathways	14	**-1.82**	4.09e-03	4.61e-02	*Ppp2r5a, Ppp2r2a, Phkb, Ppp2ca, Gbe1, Phkg2, Ugp2, Agl*
Propanoate Metabolism	KEGG	27	**-1.81**	1.67e-03	1.45e-02	*Dbt, Echdc1, Acaca, Hadha, Dld, Suclg1, Acacb, Acads*
Reactive Oxygen Species Pathway	Hallmark	25	**-1.81**	1.27e-03	4.69e-03	*Sod1, Txnrd2, Mgst1, Msra, Gclm, Prdx6, Gclc, Prdx4*
Omega-3/Omega-6 Fatty Acid Synthesis	WikiPathways	11	**-1.81**	3.24e-03	4.27e-02	*Elovl2, Acox3, Acsl1, Fads1, Fads2*
Eicosanoid Metabolism Via LOX	WikiPathways	11	**-1.80**	3.44e-03	4.27e-02	*Lta4h, Ptgr2, Cyp4f15, Ehhadh, Acox3, Acaa1a, Cyp4f14, Hpgd*
Eicosanoid Metabolism Via COX	WikiPathways	11	**-1.80**	3.44e-03	4.27e-02	*Ptgr2, Cyp4f15, Ehhadh, Acox3, Acaa1a, Cyp4f14, Hpgd*
Beta-Alanine Metabolism	KEGG	20	**-1.80**	1.41e-03	1.32e-02	*Aldh7a1, Acads, Hibch, Dpyd, Cndp2, Abat, Ehhadh, Acox3*
Chemical Carcinogenesis - Receptor Activation	KEGG	66	**-1.80**	3.59e-04	3.96e-03	*Gstm2, Gstt1, Gstm7, Akt2, Gstt3, Gstm3, Mapk3, Mgst1*
Linoleic Acid Metabolism	KEGG	16	**-1.79**	3.20e-03	2.60e-02	*Cyp2c70, Cyp3a25, Cyp2c54, Cyp2c67, Cyp2c68, Cyp2c37, Cyp2j6, Cyp2c50*
Omega-9 Fatty Acid Synthesis	WikiPathways	11	**-1.78**	4.49e-03	4.61e-02	*Elovl6, Fasn, Elovl2, Scd1, Acsl1, Fads1, Fads2*
Tamoxifen Metabolism	WikiPathways	13	**-1.78**	4.36e-03	4.61e-02	*Cyp2d22, Ugt2b35, Ugt2b1, Cyp1a2, Cyp2a5, Ugt2b36, Fmo1*
Glutathione Metabolism	KEGG	40	**-1.77**	1.46e-03	1.32e-02	*Gstp1, Gstm1, Gpx4, Gsto1, Gstm2, Gstt1, Nat8, Gpx1*
DNA Damage Response Only ATM Dependent	WikiPathways	21	**-1.75**	5.32e-03	4.90e-02	*Akt2, Scp2, Mapk9, Mapk1, Ldlr, Cat*
Tryptophan Metabolism	WikiPathways	22	**-1.75**	5.30e-03	4.90e-02	*Dld, Got2, Haao, Gcdh, Acmsd, Aldh8a1, Ido2, Ddc*
Glycerolipid Metabolism	KEGG	18	**-1.73**	7.21e-03	5.01e-02	*Abhd16a, Aldh7a1, Glyctk, Akr1a1, Gpam, Mgll, Agpat2, Aldh3a2*
Pantothenate And CoA Biosynthesis	KEGG	13	**-1.73**	5.02e-03	3.60e-02	*Dpyd, Enpp3, Coasy, Aldh3a2, Dpys*
Bempedoic Acid Therapy In Atherosclerosis	WikiPathways	11	**-1.72**	9.51e-03	7.53e-02	*Prkag1, Prkaa2, Acaca, Acly, Acacb, Prkab1, Mapk3, Mapk9*
Lipid Metabolism Pathway	WikiPathways	16	**-1.72**	7.61e-03	6.58e-02	*Prkag1, Prkaa2, Acaca, Acly, Prkab1, Fasn, Akt2, Bckdha*

Pathway Name	Database	Gene Set Size	NES	P-value	FDR q-value	Leading Edge Genes
Cholestasis	WikiPathways	10	**-1.71**	8.23e-03	6.70e-02	*Slc10a1, Abcb4, Abcb11, Ldlr, Slc22a1, Ephx1*
Fluoropyrimidine Activity	WikiPathways	13	**-1.70**	1.08e-02	8.15e-02	*Dpyd, Upp2, Ces1d, Dpys, Abcg2, Cyp2a5*
NRF2 Pathway	WikiPathways	51	**-1.70**	2.20e-03	3.48e-02	*Slc2a2, Gsta3, Gclm, Ugt1a1, Prdx6, Gclc, Ces1d, Slc2a9*
Progesterone-Mediated Oocyte Maturation	KEGG	15	**-1.70**	9.71e-03	6.38e-02	*Akt2, Mapk3, Prkacb, Mapk9, Mapk1, Hsp90aa1, Hsp90ab1*
Longevity Regulating Pathway	KEGG	20	**-1.68**	5.03e-03	3.60e-02	*Akt1s1, Prkag1, Prkaa2, Hspa8, Prkab1, Sod1, Akt2, Hspa1b*
Fatty Acid Degradation	KEGG	39	**-1.67**	3.57e-03	2.80e-02	*Aldh7a1, Acads, Gcdh, Eci1, Adh1, Acadsb, Acsl5, Ehhadh*
Nuclear Receptors Metapathway	WikiPathways	103	**-1.64**	2.40e-03	3.61e-02	*Gstm7, Slco2b1, Fasn, Apoc3, Mgst1, Baat, Slc10a1, Abcb4*
Histidine Metabolism	KEGG	14	**-1.63**	1.20e-02	7.50e-02	*Hal, Aldh9a1, Maob, Amdhd1, Aldh7a1, Cndp2, Aldh3a2, Uroc1*
Cholesterol Biosynthesis Pathway In Hepatocytes	WikiPathways	65	**-1.59**	5.32e-03	4.90e-02	*Akr1d1, Baat, Acot8, Sc5d, Abcb11, Hsd17b2, Elovl2, Amacr*
Carbon Metabolism	KEGG	84	**-1.47**	1.29e-02	7.82e-02	*Me1, Agxt2, Eno1, Sdha, Sdhc, Gldc, Pgk1, Pfkl*
Chemical Carcinogenesis - Reactive Oxygen Species	KEGG	119	**-1.40**	1.40e-02	8.28e-02	*Cox5a, Gstm2, Gstt1, Sod1, Gstm7, Uqcrc2, Akt2, mtAtp6*

NES, normalized enrichment score; FDR, false discovery rate. Pathways with FDR < 0.25 considered significant. Pathways discussed in main text. Leading edge genes shown are the top contributors to enrichment.

Table S3. Characteristics of eICU-CRD patients with versus without a serum triglyceride measurement

Characteristic	With TG measurement (n = 22,727)	Without TG measurement (n = 94,399)	SMD	P-value
Age, years	63.9 ± 15.8	64.1 ± 17.0	-0.013	<0.001
Male sex	12,899 (56.8)	50,419 (53.4)	0.067	<0.001
Body mass index, kg/m²	29.6 ± 8.5	29.1 ± 8.5	0.049	<0.001
APACHE IV score	58.1 ± 26.7	57.1 ± 25.1	0.038	0.058
Sepsis	3,541 (15.6)	15,254 (16.2)	-0.016	0.034
Surgical admission	2,795 (12.3)	13,753 (14.6)	-0.067	<0.001
Acute kidney injury (any stage)	5,956 (26.2)	18,949 (20.1)	**0.146**	<0.001
Baseline creatinine, mg/dL	1.3 ± 1.2	1.3 ± 1.3	-0.010	<0.001
Peak creatinine, mg/dL	1.6 ± 1.6	1.5 ± 1.4	0.064	<0.001
Renal replacement therapy	769 (3.4)	1,939 (2.1)	0.082	<0.001
Diabetes mellitus	6,863 (30.2)	27,990 (29.7)	0.012	0.107
ICU length of stay, days	5.3 ± 6.5	3.4 ± 3.9	**0.342**	<0.001
In-hospital mortality	2,394 (10.5)	7,780 (8.2)	0.079	<0.001

Data are presented as mean ± standard deviation for continuous variables and as number (percentage) for categorical variables. Patients comprise the eICU-CRD analytic cohort (N = 117,126) classified by whether at least one post-admission serum triglyceride (TG) measurement was available. Standardized mean differences (SMD) quantify between-group imbalance; values ≥ 0.10 (shown in bold) indicate a potentially meaningful difference. P-values are from the Mann–Whitney U test (continuous variables) and the χ^2 test (categorical variables); owing to the large sample size, P-values are significant even for negligible differences, so the SMD is the more informative measure of imbalance. Patients with a TG measurement had a longer ICU length of stay (SMD = 0.341) and a higher prevalence of acute kidney injury (26.2% vs 20.1%; SMD = 0.145), indicating that serum lipids were measured selectively in sicker patients; all other characteristics were well balanced (SMD < 0.10).
Abbreviations: AKI, acute kidney injury; APACHE IV, Acute Physiology and Chronic Health Evaluation IV; eICU-CRD, eICU Collaborative Research Database; ICU, intensive care unit; SMD, standardized mean difference; TG, triglyceride.

Table S4. Antibodies used in this study

Target Protein	Host Species	Clonality	Vendor	Catalog Number	RRID	Dilution (WB)	Dilution (IHC)	Application
GRP78/BiP	Rabbit	Monoclonal	Cell Signaling Technology	#3177	AB_2119845	1:1000	—	WB
CHOP/DDIT3	Mouse	Monoclonal	Cell Signaling Technology	#2895	AB_2089254	1:1000	—	WB
ApoB	Rabbit	Polyclonal	Abcam	ab20737	AB_449929	1:1000	—	WB
TLR4	Rabbit	Monoclonal	Cell Signaling Technology	#14358	AB_2798529	1:1000	—	WB
MyD88	Rabbit	Polyclonal	Cell Signaling Technology	#4283	AB_10547882	1:1000	—	WB
CD68	Rat	Monoclonal	Abcam	ab53444	AB_869007	—	1:200	IHC
F4/80	Rat	Monoclonal	Abcam	ab11110	AB_297810	—	1:200	IHC
β-Actin	Mouse	Monoclonal	Cell Signaling Technology	#3700	AB_2242334	1:5000	—	WB
GAPDH	Rabbit	Monoclonal	Cell Signaling Technology	#2118	AB_561053	1:5000	—	WB

WB, Western blot; IHC, immunohistochemistry; RRID, Research Resource Identifier. RRIDs were obtained from the Antibody Registry (antibodyregistry.org).

Table S5. Primer sequences used for quantitative real-time PCR

Gene Symbol	Forward Primer (5'→3')	Reverse Primer (5'→3')
Hspa5	ACTTGGGGACCACCTATTCCT	ATCGCCAATCAGACGCTCC
Ddit3	CTGCCTTTCACCTTGGAGAC	CGTTTCCTGGGGATGAGATA
Xbp1s	CTGAGTCCGAATCAGGTGCAG	GTCCATGGGAAGATGTTCTGG
Xbp1t	AAGAACACGCTTGGGAATGG	ACTCCCCTTGGCCTCCAC
Apob	GCCCATTGTGGACAAGTTGATC	CCAGGACTTGGAGGTCTTGGA
Tnf	CCCTCACACTCAGATCATCTTCT	GCTACGACGTGGGCTACAG
Il1b	GCAACTGTTCCTGAACTCAACT	ATCTTTTGGGGTCCGTCAACT
Il6	TAGTCCTTCCTACCCCAATTTCC	TTGGTCCTTAGCCACTCCTTC
Mttp	CCAGGAAAGGTTCCTCTATGCC	GACTCTCTGATGTCGTTGCTTGC
Cd14	TTGAACCTCCGCAACGTGTCGT	CGCAGGAAAAGTTGAGCGAGTG
Md-2	CTGAACCCTGCATAAGACTGAGG	CTTCCTTACGCTTCGGCAACTC
F4/80	CGTGTTGTTGGTGGCACTGTGA	CCACATCAGTGTTCCAGGAGAC
Cd68	GGCGGTGGAATACAATGTGTCC	AGCAGGTCAAGGTGAACAGCTG
Cd163	GGCTAGACGAAGTCATCTGCAC	CTTCGTTGGTCAGCCTCAGAGA
Fabp1	AGGAGTGCGAACTGGAGACCAT	GTCTCCATTGAGTTCAGTCACGG
Acox1	GCCATTCGATACAGTGCTGTGAG	CCGAGAAAGTGGAAGGCATAGG
GAPDH	AGGTCGGTGTGAACGGATTTG	TGTAGACCATGTAGTTGAGGTCA

All primers designed for Mus musculus (mouse).

Table S6. Covariate balance before and after propensity-score matching in the MIMIC-IV cohort.

Matching covariate	SMD before matching	SMD after matching
Age	0.143	0.037
Male sex	0.047	0.009
Non-renal SOFA score	0.593	0.010
Sepsis	0.383	0.016
Diabetes	0.145	0.024
Vasopressor use	0.572	0.013
Emergency admission	0.047	0.034
Other admission category	0.110	0.018
Surgical admission	0.083	0.016

Propensity scores were estimated by logistic regression (scikit-learn) and matched 1:1 by nearest-neighbor matching without replacement (caliper = 0.2 standard deviations of the logit-transformed propensity score, = 0.1399), yielding 3,498 matched pairs (6,996 patients). Standardized mean differences (SMD) < 0.10 indicate adequate balance. All nine matching covariates achieved adequate balance after matching; the maximum SMD decreased from 0.593 (non-renal SOFA score) before matching to 0.037 (age) after matching. Admission category was modeled as indicator variables with elective admission as the reference. **Abbreviations:** MIMIC-IV, Medical Information Mart for Intensive Care IV; SMD, standardized mean difference; SOFA, Sequential Organ Failure Assessment.
